# Enhancement of Photoluminescence Quantum Yield of Silver Clusters by Heavy Atom Effect

**DOI:** 10.1002/smll.202500700

**Published:** 2025-03-03

**Authors:** Aoi Akiyama, Sakiat Hossain, Yoshiki Niihori, Kazutaka Oiwa, Jayoti Roy, Tokuhisa Kawawaki, Thalappil Pradeep, Yuichi Negishi

**Affiliations:** ^1^ Department of Chemistry Graduate School of Science Tokyo University of Science 1−3 Kagurazaka, Shinjuku‐ku Tokyo 162−8601 Japan; ^2^ Research Institute for Science & Technology Tokyo University of Science 1−3 Kagurazaka, Shinjuku‐ku Tokyo 162−8601 Japan; ^3^ DST Unit of Nanoscience (DST UNS) and Thematic Unit of Excellence (TUE) Department of Chemistry Indian Institute of Technology Madras Chennai 600036 India; ^4^ Institute of Multidisciplinary Research for Advanced Materials Tohoku University 2−1−1 Katahira, Aoba‐ku Sendai 980−8577 Japan

**Keywords:** anion templating, heavy atom effect, ligand‐protected clusters, phosphorescence, precise synthesis

## Abstract

Many ligand‐protected metal clusters exhibit phosphorescence at room temperature. However, strategies for improving their phosphorescence quantum yield, a critical parameter of performance, remain poorly developed. In contrast, fluorescent dyes are commonly modified by introducing heavy atoms, such as iodine (I), to enhance intersystem crossing in the excited state, thereby harnessing the heavy atom effect to increase phosphorescence efficiency. In this study, a pair of ligand‐protected silver (Ag) clusters is successfully synthesized with internal cavities encapsulating anions (X*^z^* ^−^), namely sulfide ions (S^2−^) or iodide ions (I^−^), which significantly differ in atomic number each other. Single‐crystal X‐ray diffraction and nuclear magnetic resonance spectroscopy revealed that the resulting Ag clusters are composed of X@Ag_54_S_20_(thiolate)_20_(sulfonate)*_m_*, where (X, *m*) = (S, 12) or (I, 11). X‐ray photoelectron spectroscopy revealed that the Ag atoms in these compounds exhibit a mixed‐valence state. Furthermore, experiments on their photoluminescence revealed that a heavy central anion induced an internal heavy‐atom effect similar to that observed in organic fluorescent dyes. As a result, the phosphorescence quantum yield became 16 times higher when S^2−^ is replaced by I^−^ as the central atom.

## Graphical abstract

Novel Ag_54_ clusters, composed of silver (Ag) and sulfur and protected by thiolate and sulfonate, has a large internal cavity that can encapsulate sulfide and iodide ions. The encapsulation of iodide ions into Ag_54_ enhances the spin‐orbit interaction and improves phosphorescence properties.

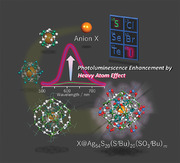

## 1 Introduction

Progress in nanotechnology is facilitated by the development of precise synthesis methods and the detailed characterization of fine and sophisticated nanomaterials. Ligand‐protected metal clusters, a representative group of nanomaterials, have been the focus of numerous studies, resulting in the establishment of precise synthesis methods and the elucidation of their properties through synergistic experimental and theoretical investigations.^[^ ^1^ ^]^ Among these, ligand‐protected metal clusters composed of noble metal elements, such as gold (Au), silver (Ag), and copper (Cu), exhibit unique properties according to the number of constituent atoms and dopant elements, including photoluminescence (PL),^[^ ^2^ ^]^ magnetic properties,^[^ ^3^ ^]^ and catalytic activity.^[^ ^4^ ^]^ These characteristics have attracted significant attention in both fundamental research and practical applications.^[^ ^5^ ^]^ Recently, studies have also been conducted on clusters with complex structures arising from combinations of noble metal elements and main group elements.^[^ ^6^ ^]^ Among these, Ag–sulfur (Ag─S) based clusters are metal clusters with cavities^[^ ^7^ ^]^ capable of encapsulating various anions,^[^ ^8^ ^]^ and recent efforts have focused on altering the encapsulated anionic species to modulate the physicochemical properties of these clusters.^[^ ^7^, ^9^ ^]^

Ligand‐protected metal clusters often exhibit PL.^[^ ^10^ ^]^ Recent experimental studies have shown that phosphorescence originates in the excited triplet state.^[^ ^11^ ^]^ Therefore, enhancing the population of the excited triplet state of metal clusters is crucial for their application as room‐temperature phosphorescent materials and triplet sensitizers.^[^ ^12^ ^]^ Generally, transition from the excited singlet state (S*_n_*) to the excited triplet state (T*_m_*) is spin‐forbidden, resulting in a zero transition‐moment. However, studies on organic fluorescent dyes have shown that introducing heavy atoms, such as iodine (I), into the fluorophore enhances spin‐orbit coupling (the internal heavy‐atom effect), which increases the rate constant for intersystem crossing (ISC) from S*_n_* to T*_m_*, thereby improving the efficiency of phosphorescence.^[^ ^13^ ^]^ Similarly, encapsulating a heavy atom into Ag─S clusters may enhance the phosphorescence quantum yield. However, to date, there are limited reports on the internal heavy‐atom effect of ligand‐protected metal clusters.^[^ ^14^ ^]^

**Scheme 1:**
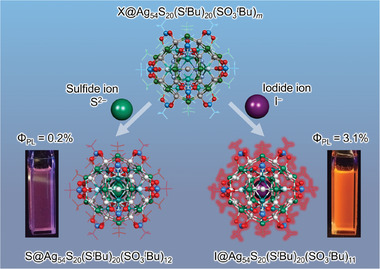
Enhancement of phosphorescence quantum yield by encapsulating S^2−^ or I^−^ anions inside the central cavity of the Ag_54_S_20_(S*^t^* Bu)_20_(SO_3_ *^t^* Bu)*_m_* framework.

In this study, we successfully synthesized ligand‐protected Ag─S clusters with central cavities encapsulating anions X*^z^* ^−^, where X*^z^* ^−^ = S^2−^ (sulfide) or I^−^ (iodide), which significantly differ in atomic number each other (**Scheme** **1**). Single‐crystal X‐ray diffraction (SC‐XRD) and proton nuclear magnetic resonance (^1^H NMR) spectroscopy revealed that the resulting Ag clusters were composed of X@Ag_54_S_20_(thiolate)_20_(sulfonate)*_m_*, where (X, *m*) = (S, 12) or (I, 11) **(X@Ag54)**. Furthermore, a comparison of the optical properties of the obtained pair of **X@Ag54** (X = S or I) demonstrated that an internal heavy‐atom effect occurred in these Ag–S clusters, similar to that in organic fluorescent dyes. Accordingly, the phosphorescence quantum yield was 16 times higher when S^2−^ was replaced by I^−^ as the encapsulated atom (Scheme 1).

## 2 Results and Discussion

### 2.1 Synthesis

For the synthesis of **S@Ag54**, 48.6 mg (0.22 mmol) of silver trifluoroacetate (Ag(TFA)) and 10.6 mg (0.044 mmol) of copper(II) nitrate trihydrate (Cu(NO_3_)_2_∙3H_2_O) were dissolved in 4 mL of a mixture of acetone and acetonitrile (50/50 vol%). Then, 15 µL (0.13 mmol) of *tert*‐butanethiol (*^t^* BuSH) was added to this solution, and the resulting mixture was transferred into a glass tube (Figure S1A, Supporting Information). A cap with a small hole (Figure S1B, Supporting Information) was placed on the glass tube, and the tube was left undisturbed under a LED for light irradiation to promote copper‐catalyzed thiol oxidation (Figure S1C, Supporting Information). Orange crude crystals of **S@Ag54** were obtained after ≈1 week (Figure S2Aa, Supporting Information). The crude crystals were dissolved in chloroform, and hexane was slowly added to the upper layer. The solution was left to stand for one month, resulting in the formation of single crystals of **S@Ag54** (Figure S2Ba, Supporting Information).

**I@Ag54** was synthesized in the same manner as **S@Ag54**, except 20 mg (0.054 mmol) of tetrabutylammonium iodide (TBAI), as well as *^t^* BuSH, was added to the acetone/acetonitrile mixture of Ag(TFA)/Cu(NO_3_)_2_. Red‐orange crude crystals of **I@Ag54** were obtained after ≈2 weeks (Figure S2Ab, Supporting Information). The crude crystals were dissolved in chloroform, and hexane was slowly added to the upper layer. The solution was left to stand for one month, resulting in the formation of single crystals of **I@Ag54** (Figure S2Bb, Supporting Information).

### 2.2 Chemical Composition

**Figure 1:**
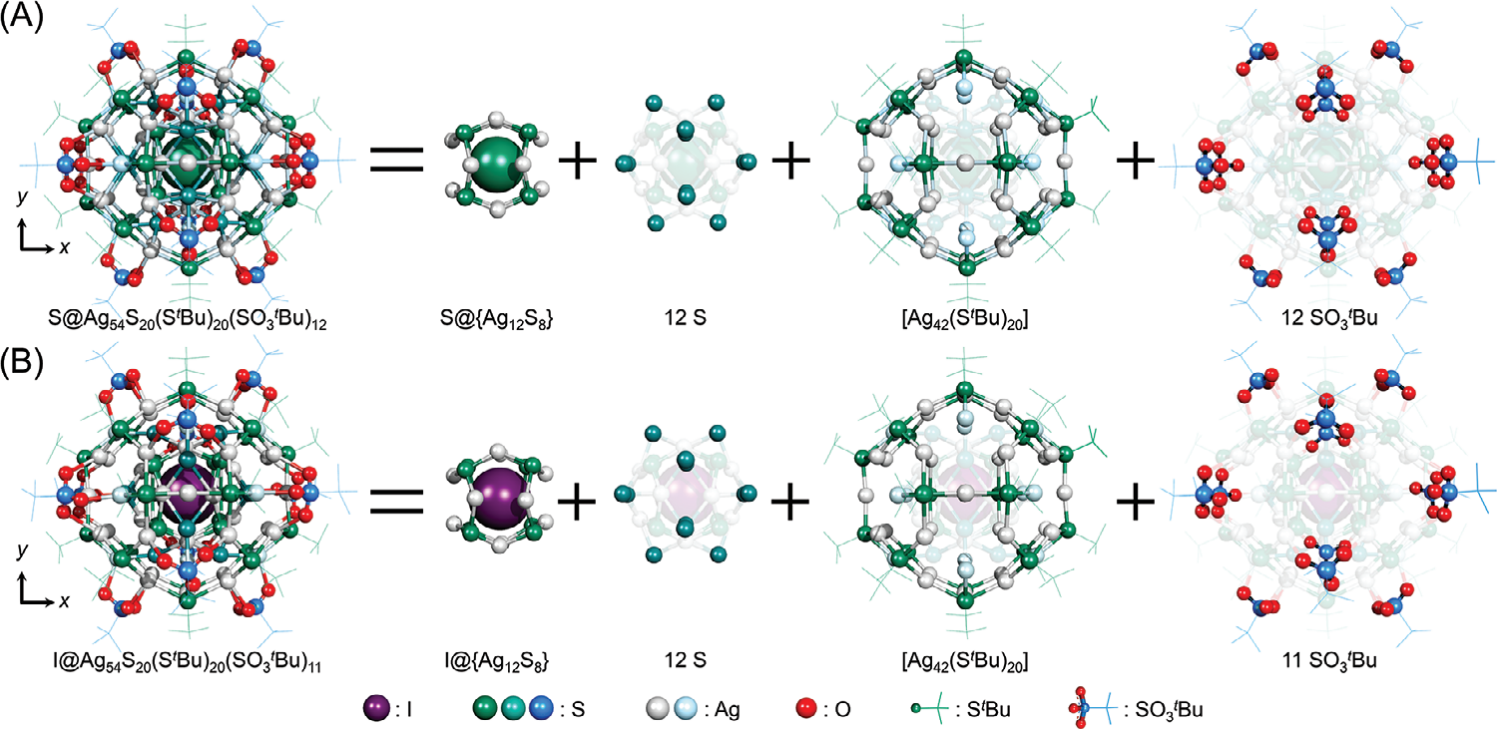
Geometric structures of A) **S@Ag54** and B) **I@Ag54**. The distances between the central atoms (S or I) and the nearest Ag atoms in **S@Ag54** and **I@Ag54** are 3.19 and 3.27 Å, respectively (Figure S5, Supporting Information). Considering the ionic radius, **S@Ag54** can be considered to have no bond between S and the Ag atoms of core. However, this distance is shorter than the total value of van der Waals radius of S and Ag atoms. Therefore, it can be considered that a weak bond exists also in the core of **S@Ag54**.

**Figure 2:**
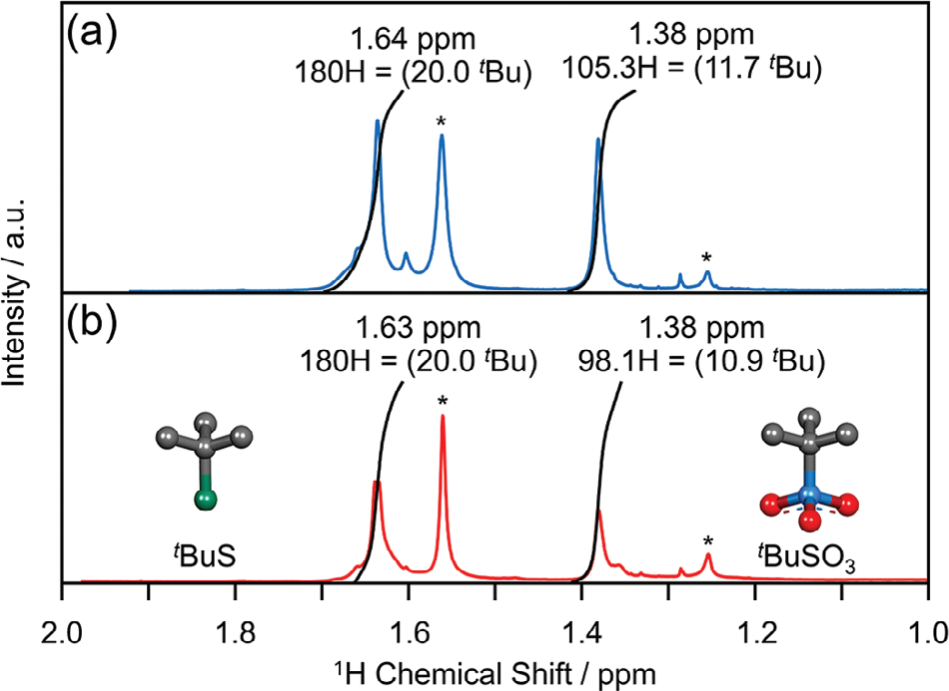
^1^H NMR spectra of a) **S@Ag54** and b) **I@Ag54** in CDCl_3_. The asterisks (^*^) mark the peaks attributed to impurities, such as water (1.56 ppm) or alkanes/grease (1.25 ppm).

**S@Ag54** and **I@Ag54** were crystallized in space groups of *Fm*−3 and *Pa*−3, respectively (Table S1, Supporting Information), and their geometric structures were determined using SC‐XRD (**Figure** **1**A,B). According to these geometric structures, the chemical compositions of **S@Ag54** and **I@Ag54** were X@Ag_54_S_20_(S*^t^* Bu)_20_(SO_3_ *^t^* Bu)*_m_* (X = S and I; S*^t^* Bu = *tert*‐butanethiolate; SO_3_ *^t^* Bu = *tert*‐butyl sulfonate) (hereafter, **X@Ag54**). Although the number of S*^t^* Bu and SO₃*^t^* Bu ligands for both **S@Ag54** and **I@Ag54** were estimated by SCXRD to be 20 and 12, respectively, **S@Ag54** and **I@Ag54** may have different ligand numbers because S and I have different families on the periodic table. Therefore, an attempt was made to estimate the number of ligands in **I@Ag54** through ^1^H NMR analysis. In the ^1^H NMR spectra of **S@Ag54** and **I@Ag54** (**Figure** **2**), the two peaks at 1.63 and 1.38 ppm can be attributed to the protons of S*^t^* Bu and SO_3_ *^t^* Bu, respectively.^[^ ^6b,h^ ^]^ The integral values of these peaks revealed that **S@Ag54** contained 20 S*^t^* Bu and 12 SO_3_ *^t^* Bu ligands, while **I@Ag54** contained 20 S*^t^* Bu and 11 SO_3_ *^t^* Bu ligands. These results indicated that the chemical compositions of **S@Ag54** and **I@Ag54** were S@Ag_54_S_20_(S*^t^* Bu)_20_(SO_3_ *^t^* Bu)_12_ and **I@Ag_54_** S_20_(S*^t^* Bu)_20_(SO_3_ *^t^* Bu)_11_, respectively (Figure 1).

The above chemical compositions indicate that there is a difference in the number of SO_3_ *^t^* Bu ligand between **S@Ag54** and **I@Ag54**. Ligand‐protected metal clusters are generally formed when the total number of valence electrons is even.^[^ ^6^, ^15^ ^]^ In this study, the geometrical structure obtained by SC‐XRD and electrospray ionization (ESI) mass spectrum at the low mass region showed that TFA^−^, which could be a counter ion, was not observed for either **S@Ag54** or **I@Ag54**, suggesting that these clusters were neutral. In this case, because S@Ag_54_S_20_(S*^t^* Bu)_20_(SO_3_ *^t^* Bu)_12_ has an even number of valence electrons, **I@Ag54** with the same ligand combination (I@Ag_54_S_20_(S*^t^* Bu)_20_(SO_3_ *^t^* Bu)_12_) should have an odd number of valence electrons. Therefore, it can be considered that **I@Ag54** was formed with one fewer SO_3_ *^t^* Bu ligand than **S@Ag54** to prevent destabilization due to the odd number of valence electrons.

To gain a deeper understanding of the chemical composition, we also acquired ESI mass spectra of the products. The ESI‐mass spectra of both **S@Ag54** and **I@Ag54** contained multiple peaks attributed to ions in which AgS or ligands were adsorbed or desorbed from the clusters^[^ ^16^ ^]^ (e.g., **I@Ag54** + 2 AgS) (Figure S3 and Tables S2 and S3, Supporting Information). These results confirmed that the estimation of the above chemical compositions is correct for both **X@Ag54**.

### 2.3 Geometric Structure

The geometric structures of **X@Ag54** clusters were similar each other, with both containing an X@{Ag_12_S_8_} core (Figure 1A,B). In this core, the central anion X (S^2−^ or I^−^) was surrounded by 12 Ag atoms, forming an icosahedron (point group *I*_h_) (Figure S4, Supporting Information), with 8 S atoms in a cubic arrangement surrounding this core. On the surface of the X@{Ag_12_S_8_} core, 12 S atoms formed an intermediate layer (highlighted in yellow, Figure S4A, Supporting Information), which was connected to outer Ag_42_(S*^t^* Bu)_20_. The 12 S atoms of the intermediate layer were bonded to 12 Ag atoms of Ag_42_(S*^t^* Bu)_20_ (X@{Ag_12_S_8_}S_12_[Ag_42_(S*^t^* Bu)_20_]) (highlighted in blue, Figure S4A, Supporting Information). On the surface of X@{Ag_12_S_8_}S_12_[Ag_42_(S*^t^* Bu)_20_], the S atoms of S(*^t^* Bu) were arranged in a regular icosahedral structure with two coordination modes: μ_3_‐S(*^t^* Bu) and μ_4_‐S(*^t^* Bu) (Figure S4B,C, Supporting Information). The *m* SO_3_ *^t^* Bu ligands were coordinated with 12 Ag atoms of Ag_42_(S*^t^* Bu)_20_ (highlighted in blue, Figure S4A, Supporting Information), which were bonded to the S atoms of the intermediate layer, as well as 2 Ag atoms of remaining Ag_30_(S*^t^* Bu)_20_ (highlighted in red, Figure S4A, Supporting Information) in the coordination mode of μ_3_‐O_3_S(*^t^* Bu)‐κ^3^O, Oʹ, Oʹʹ (Figure S4D, Supporting Information). Owing to this structure, **X@Ag54** can be described as X@{Ag_12_S_8_}S_12_[Ag_42_(S*^t^* Bu)_20_(SO_3_ *^t^* Bu)*_m_*].

In these structures, both the X@{Ag_12_S_20_} part and surrounding Ag_42_(S*^t^* Bu)_20_(SO_3_ *^t^* Bu)*_m_* shell exhibit the same *T*_h_ symmetry. However, as mentioned earlier, **I@Ag54** has one fewer SO_3_ *^t^* Bu ligand than **S@Ag54**. The SC‐XRD results suggest that a SO_3_ *^t^* Bu ligand is missing from a random site, rather than a specific site, in **I@Ag54**. Therefore, in **I@Ag54**, the 12 coordination sites are randomly occupied by 11 SO_3_ *^t^* Bu ligands, leading to the formation of multiple structural isomers and accordingly, disorder in the geometric structure of **I@Ag54**.

These geometric structures of **X@Ag54** are similar to those of previously reported clusters, such as S@Ag_40.13_Cu_13.87_S_18_(S*^t^* Bu)_20_(SO_3_ *^t^* Bu)_12_ and S@Cu_54_S_12_O_6_(S*^t^* Bu)_20_(SO_3_ *^t^* Bu)_12_.^[^ ^6h,i^ ^]^ In the two previously reported clusters, the M_42_(S*^t^* Bu)_20_(SO_3_ *^t^* Bu)_12_ (M = Ag or Cu) shell, which has *T*_h_ symmetry, also covers the core. However, there are two major differences between these two previously reported clusters and **X@Ag54**: 1) in the previously reported clusters, Ag and Cu, or only Cu, are included as metals, whereas in **X@Ag54**, only Ag is present as the metal of the clusters; 2) the previously reported clusters have two fewer chalcogenide ions (S^2−^ or O^2−^) other than the central ion (18 chalcogenide ions) than **X@Ag54** (20 sulfide ions). As a result, the diameter of the cavity of the M_12_S_8_ core of **X@Ag54** (≈5.04 and 5.13 Å for **S@Ag54** and **I@Ag54**, respectively; Figures S4E and S5, Supporting Information), is greater than that of the two previously reported clusters (4.94 and 4.88 Å).^[^ ^6^, ^17^ ^]^ For this reason, both S^2−^ (ionic radius = 1.84 Å) and I^−^ (ionic radius = 2.20 Å),^[^ ^18^ ^]^ were stably encapsulated inside the {M_12_S_8_} core of **X@Ag54**.

### 2.4 Formation Mechanism

**Figure 3:**
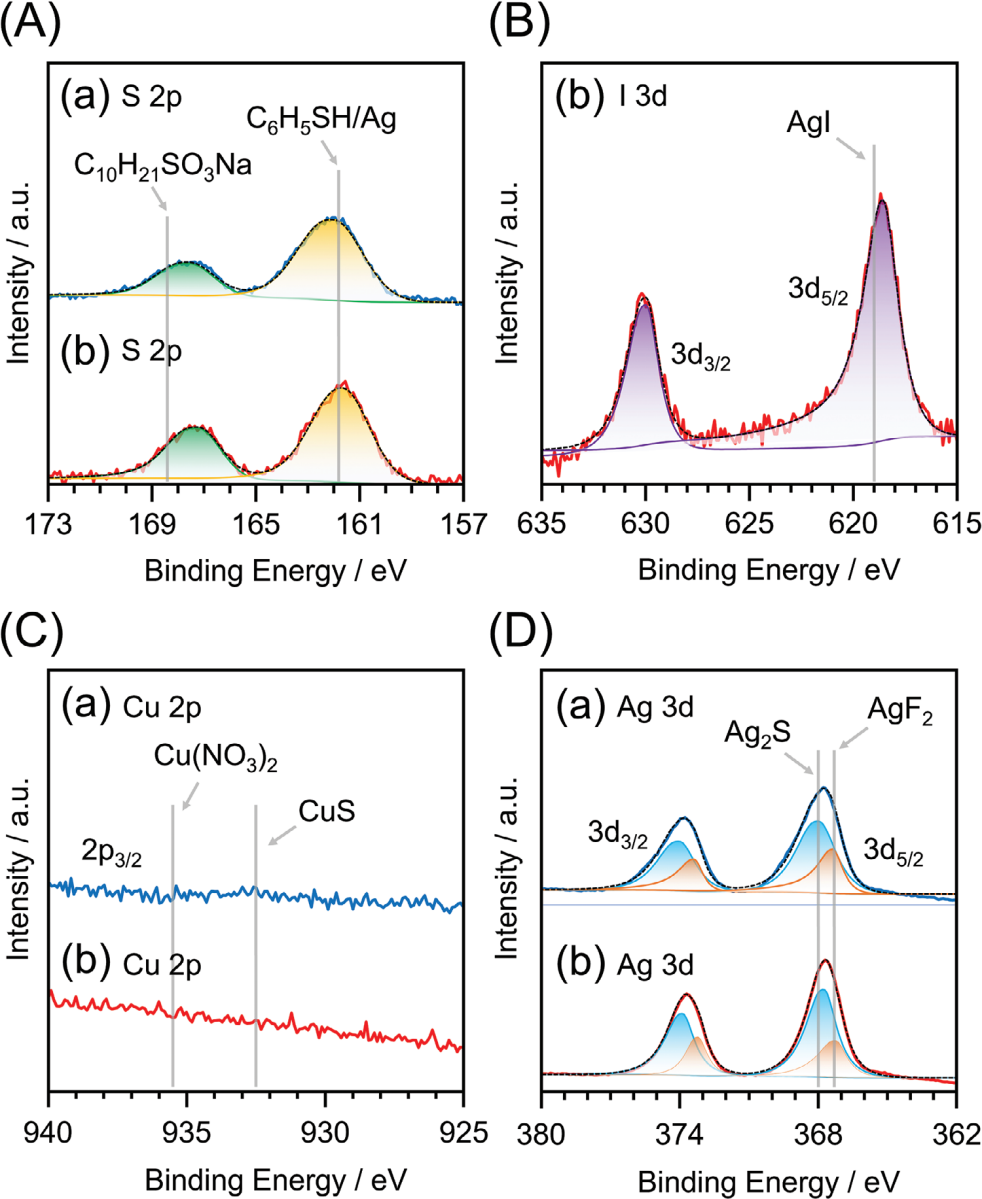
X‐ray photoelectron spectra of A) S 2p, B) I 3d, C) Cu 2p, D) and Ag 3d for (a) **S@Ag54** and (b) **I@Ag54**. Different colors of fitting curves in spectra correspond to different oxidation states. In (A), the peaks on the low binding energy side (yellow) are attributed to sulfide S^2−^ or thiolate *^t^* BuS^−^, and the peaks on the high binding energy side (green) are attributed to S derived from sulfonate *^t^* BuSO_3_^−^. Spectrum (B) indicates that the charge state of I is similar to that of I^−^. Spectrum (C) indicates that **X@Ag54** does not include Cu. In (D), the peaks ≈368 and 374 eV are attributed to Ag 3d_5/2_ and Ag 3d_3/2_, respectively. These spectra indicated that the oxidation state of Ag in **X@Ag54** was similar to that of Ag_2_S and AgF_2_.

**X@Ag54** contains 20 S atoms. These S atoms are considered to be produced through the reaction between Ag─S*^t^* Bu and Ag^+^: this reaction produces Ag_2_S.^[^ ^6a^ ^]^ In this study, although only *^t^* BuSH was added as the ligand during the synthesis, in situ produced SO_3_ *^t^* Bu was also included as a ligand in **X@Ag54**, as confirmed by SC‐XRD (Figure 1), ^1^H NMR (Figure 2), and ESI‐mass spectroscopy (Figure S3, Supporting Information), in addition to X‐ray photoelectron spectroscopy (XPS) (**Figure** **3**; Figures S6, and S7, Supporting Information) and Fourier‐transform infrared (FT‐IR) absorption spectroscopy (Figure S8, Supporting Information), of the products. The catalytic oxidation of thiols by Cu^2+^ (Scheme S1, Supporting Information) is responsible for the formation of these SO_3_ *^t^* Bu ligands.^[^ ^6h,i^ ^]^ In this reaction, Cu^2+^ coordinates with RSH, which reduces Cu (Cu^+^) and generates the thiyl radical (RS∙).^[^ ^19^ ^]^ RS∙ reacts with oxygen molecular (O_2_) and unreacted RSH in the system to produce RSO_3_H (Figure S9, Supporting Information). After coordinating with RSH, Cu^+^ is oxidized to Cu^2+^ by O_2_, returning to its original state. As these reactions proceed, SO_3_ *^t^* Bu is formed in the reaction solvent and coordinates with the metal cluster (Scheme S1, Supporting Information). In these reactions, the coordination and reduction of Cu^2+^ and formation of RSO_3_H from RS∙ proceed with rate constants of ≈10^8–10^ M^−1^s^−1^,^[^ ^19^ ^]^ which is very fast. Moreover, these reactions are faster than the oxidation of Cu^+^ to Cu^2+^ by O_2_, which becomes the rate‐limiting step. Indeed, when Cu(NO_3_)_2_ and *^t^* BuSH were mixed, the blue color of the solution immediately disappeared owing to the reduction of Cu^2+^ to colorless Cu^+^, whereas this blue color took 2 days to reappear as Cu^+^ reverted to Cu^2+^ (Figure S10Ba, Supporting Information). However, when Ag^+^ coexisted with Cu^2+^ and *^t^* BuSH, the recovery of Cu^2+^ from Cu^+^ occurred more rapidly (Figure S10, Supporting Information), with the disappearance of *^t^* BuSH confirmed by electron spin resonance (ESR) spectroscopy of the reaction solution. These results demonstrate that the presence of Ag^+^ accelerates the rate‐limiting step of RSH oxidation by Cu^2+^, thereby accelerating the formation of RSO_3_H.

Zhu et al. have reported the synthesis of S@Ag_40.13_Cu_13.87_S_18_(S*^t^* Bu)_20_(SO_3_ *^t^* Bu)_12_ containing both Ag and Cu as metals under reaction conditions similar to those used in our study.^[^ ^6h^ ^]^ However, in our study, **X@Ag54**, which contains only Ag as the metal, was synthesized (Figure 3C). In our study, unlike the research by Zhu et al., we did not add the reducing reagents, such as borane amine complexes,^[^ ^6h^ ^]^ into the reaction solvent. Moreover, compared to their study, we significantly reduced the amount of Cu^2+^ catalyst relative to Ag^+^ (1.260 vs 0.198 atom %). These factors likely contributed to the successful synthesis of **X@Ag54** with only Ag as the metal, which enabled the encapsulation of I^−^.

### 2.5 Oxidation State of Ag

The core of **X@Ag54** consisted of only Ag and S and formed a central cavity, as observed in previously reported [S@Ag_50_S_12_(S*^t^* Bu)_20_](TFA)_4_.^[^ ^6c^ ^]^ In the previously reported cluster, all the Ag atoms are in the +1 oxidation state, giving rise to a single sharp peak in both the Ag 3d_3/2_ and 3d_5/2_ regions of its XPS spectrum. In contrast, broad peaks were observed in the Ag 3d_5/2_ and 3d_3/2_ regions of the XPS spectra of **X@Ag54** (Figure 3D). These peaks were fitted with two functions, and the position of the peak on the high‐energy side was close to the position of the peak corresponding to Ag_2_S or AgF_2_ (Figure 3D; Figure S6 and Table S4, Supporting Information).^[^ ^20^ ^]^ These observations indicates that Ag in **X@Ag54** is in a mixed‐valence state, rather than the single‐valence state of Ag in [S@Ag_50_S_12_(S*^t^* Bu)_20_](TFA)_4_.^[^ ^6c^ ^]^ In fact, there have been several reports on Ag existing in mixed‐valence states.^[^ ^20^, ^21^ ^]^ Furthermore, Cu atoms in Cu–chalcogen clusters with geometric structures similar to those of **X@Ag54**, such as [S@Cu_54_S_12_O_6_(S*^t^* Bu)_20_(SO_3_ *^t^* Bu)_12_] and [Cu_50_S_12_(S*^t^* Bu)_20_(TFA)_12_], also exist in a mixed‐valence state of Cu^I^ and Cu^II^.^[^ ^6^, ^22^ ^]^ Unlike [S@Ag_50_S_12_(S*^t^* Bu)_20_](TFA)_4_, **X@Ag54** contained two types of ligands (S*^t^* Bu and SO_3_ *^t^* Bu), and this can be considered to cause the mixed‐valence state of Ag. As discussed in the (Section S3.1, Supporting Information), when **X@Ag54** has neutral charge and is 0‐electron system, the mixed‐valence states of Ag in **S@Ag54** and **I@Ag54** can be estimated to be S@Ag^I^_34_Ag^II^_20_ and I@Ag^I^_36_Ag^II^_18_, respectively. In this composition, the ratio of AgI: AgII is 62.9:37.1 and 66.7:33.3 for **S@Ag54** and **I@Ag54**, respectively. Figure 3D shows that the ratio of Ag^I^: Ag^II^ is 63.7:36.3 and 65.8:34.2 for **S@Ag54** and **I@Ag54**, respectively. In this way, the results of Ag 3d XPS will reproduce the expected mixed‐valence states of Ag for **S@Ag54** and **I@Ag54**.

### 2.6 Stability

To apply the clusters as materials, information on their stability is crucial. Therefore, the stability of **X@Ag54** against the following three factors was investigated: 1) degradation in solution, 2) heat‐induced dissociation, and 3) collision‐induced dissociation (CID).

**Figure 4:**
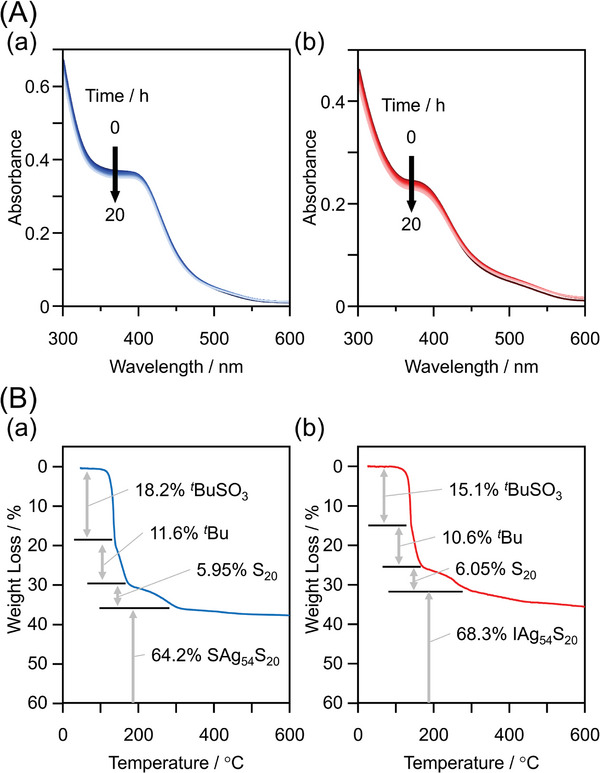
Stability of **X@Ag54**. A) Time evolution of the absorption spectrum of toluene solutions of (a) **S@Ag54** and (b) **I@Ag54** at 60 °C. B) TGA curves of (a) **S@Ag54** and (b) **I@Ag54**.

Stability against degradation in solution was evaluated by heating **X@Ag54** in toluene and tracking changes in its ultraviolet–visible (UV–vis) absorption spectrum. The results showed that 1) the stabilities of both clusters are similar, and 2) the absorption spectra of both samples in toluene can remain unchanged over 20 h of heating at 60 °C (**Figure** **4**Aa,Ab; Figure S11, Supporting Information). The half‐life of both clusters under these degradation conditions was estimated to be ≈200 h (Table S5, Supporting Information).

Stability against heat‐induced dissociation was evaluated using thermogravimetric analysis (TGA). Both clusters underwent three stages of weight loss (Figure 4Ba,Bb; Figure S12, Supporting Information). According to the weight of the ligands and their functional groups, the weight loss at ≈115 °C is attributed to the removal of SO_3_ *^t^* Bu ligands (theoretical loss = 16.6% for **S@Ag54** and 15.3% for **I@Ag54**), while the weight loss at ≈140 °C is attributed to the removal of *^t^* Bu groups from S*^t^* Bu ligands (theoretical loss = 11.5% for **S@Ag54** and 11.6% for **I@Ag54**). The final weight loss at ≈170 °C is interpreted as the removal of sulfur (theoretical loss = 6.46% for **S@Ag54** and 6.49% for **I@Ag54**). The slow weight loss at subsequent temperatures can be attributed to the desorption of sulfur or X from the remaining X@Ag_54_S_20_. The dissociation energies of Ag─SO_3_ *^t^* Bu and AgS─*^t^* Bu, calculated using density functional theory (DFT), were 54.2 and 57.4 kcal mol^−1^, respectively (Table S6, Supporting Information). This should be the reason why thermal dissociation occurred in the order. Each weight‐loss also occurred at a similar temperature. These results indicates that the stability of both clusters against thermal dissociation is similar to each other.

**Figure 5:**
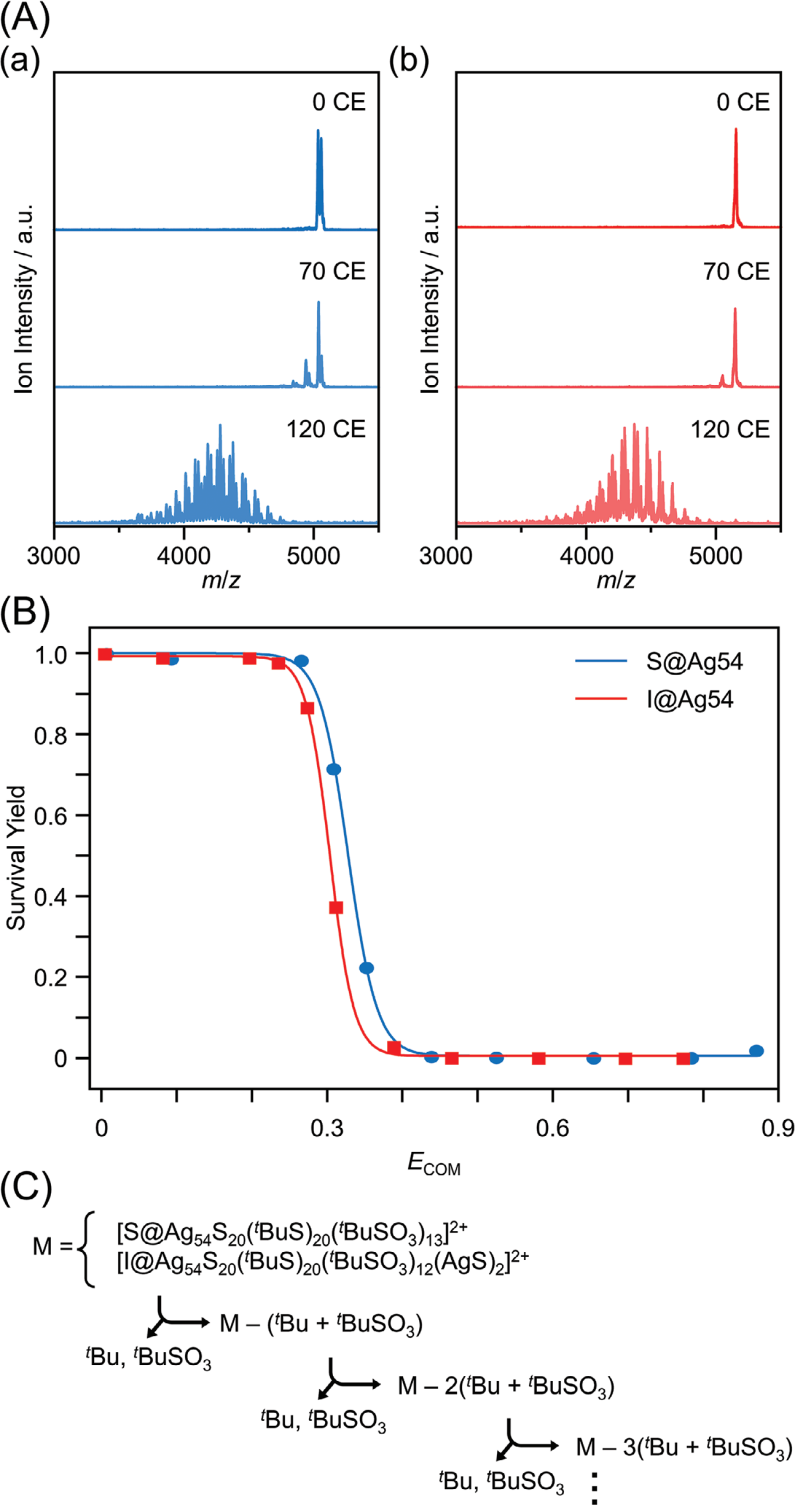
A) CID‐mass spectra of (a) **S@Ag54** and (b) **I@Ag54** as a function of collision energy. CE indicates the collision energy. B) Survival yield obtained from CID profiles of **X@Ag54** (Figure 5A). Details for calculating the survival yield are provided in the (Section S1.8, Supporting Information). C) CID process of **X@Ag54**.

Stability against CID was investigated in a vacuum.^[^ ^23^ ^]^ Specifically, **X@Ag54** was introduced into an ESI‐mass spectrometer, where it collided with argon (Ar) to undergo CID. The stability of each cluster was examined by evaluating the chemical composition and ion intensity of the generated fragment ions. Mass spectra were obtained following the collision of [**S@Ag54** + SO_3_ *^t^* Bu]^2+^ and [**I@Ag54** + SO_3_^t^Bu + 2AgS]^2+^ with Ar at different collision energies (**Figure** **5**A,B; Table S9, Supporting Information). The parent ions of **X@Ag54** dissociated as the collision energy increased. The main dissociation patterns corresponded to the radical cleavage of Ag─O_3_S*^t^* Bu and S─*^t^* Bu (Figure 5C). These dissociation patterns closely resembled those observed in the TGA curves. Furthermore, these dissociation reactions of both clusters occurred at similar collision energies (Figures S13 and S14 and Tables S7 and S8, Supporting Information), indicating that the stability of both clusters against CID was also similar to each other.

In these experiments, detachment of surface ligands was the first step of degradation. It can be considered that since the difference in the kinds of the central anions (X) do not significantly affect the binding energy between surface Ag and the ligands, the two **X@Ag54** clusters showed similar stability against the three factors tested in the above experiments.

### 2.7 Photoluminescence

**Figure 6:**
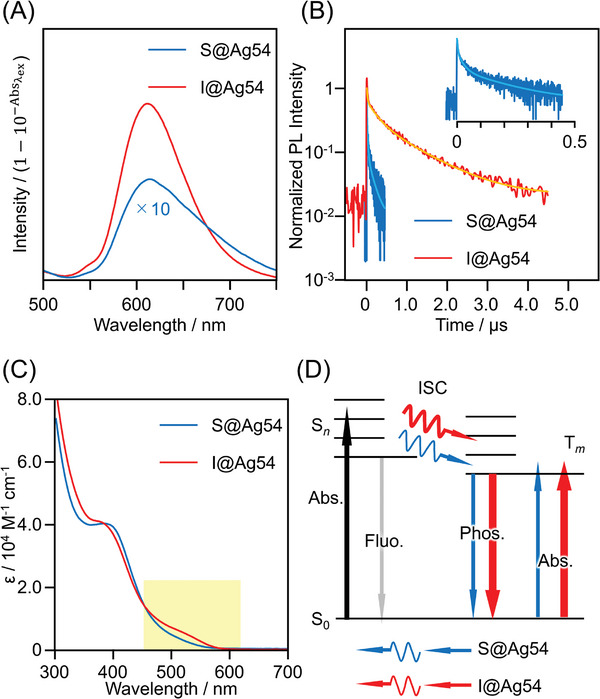
A) PL spectra of deuterated toluene solutions of **X@Ag54**. The excitation wavelength was fixed at 405 nm. The vertical axis is the PL intensity divided by the transmittance of the sample at the excitation light wavelength. B) PL decay curves of deuterated toluene solutions of **X@Ag54**. Excitation wavelength of pulse laser was fixed at 405 nm. C) UV–vis absorption spectra of toluene solutions of **S@Ag54** and **I@Ag54**. *ε* represents the molar absorption coefficient. The yellow part highlights the absorption assignable to S─T transition. D) Predicted scheme of the relaxation and absorption for **X@Ag54**.

**Figure** **6**A shows the PL spectra of **X@Ag54** in toluene under an Ar atmosphere. To obtain strong PL, the excitation wavelength was set to 405 nm. Both cluster solutions exhibited a broad PL peak at ≈610 nm (Figure S15, Supporting Information). Recent studies have shown that the PL of metal clusters consisting of a large number of metal atoms entails photoexcitation to generate a dark excited singlet state, which subsequently undergoes intersystem crossing (ISC) to a bright excited triplet state, resulting in phosphorescence.^[^ ^10^, ^11^, ^12^, ^24^ ^]^ Because **X@Ag54** also comprised a large number of metal atoms, the observed emission of **X@Ag54** is likely phosphorescence. In fact, the estimated radiative lifetime of **X@Ag54** was on the order of tens to hundreds of microseconds (**Table** **1**), which is significantly longer than that of fluorescent materials (≤1 µs).^[^ ^25^ ^]^

**Table 1:** Photoluminescence properties of **X@Ag54**.

Sample	Φ_PL_ ^a)^	*τ*_PL_ [ns]^b)^	*k*_r_ [s^−1^]^c)^	*k*_nr_ [s^−1^]^d)^	*τ*_r_ [µs]^e)^
**S@Ag54**	2.0 × 10^−4^	106.2	1.9 × 10^3^	9.4 × 10^6^	530
**I@Ag54**	3.1 × 10^−2^	652.9	4.8 × 10^4^	1.5 × 10^6^	21

^a)^ PL quantum yield;

^b)^ averaged PL lifetime;

^c)^ radiative rate constant;

^d)^ nonradiative rate constant;

^e)^ radiative lifetime (reciprocal of *k*_r_).

The photoluminescence quantum yield (Φ_PL_) of **X@Ag54** was evaluated using a relative method,^[^ ^26^ ^]^ revealing that the Φ_PL_ of **I@Ag54** was ≈16 times greater than that of **S@Ag54** (Table 1). Because there was no significant difference in the symmetry of the geometries of the two **X@Ag54** clusters, the difference in Φ_PL_ did not arise from symmetry breaking.^[^ ^27^ ^]^

However, 1) the two **X@Ag54** clusters differs in the type of anion present in their internal cavities, and 2) Φ_PL_ increases with increasing atomic number of encapsulated anion. Introducing heavy halogens (heavy atoms) into aromatic dyes, such as acene‐based compounds, enhances ISC from the excited singlet to the excited triplet state owing to the heavy atom effect, which increases the radiative rate constant (*k*_r_) and, consequently, Φ_PL_.^[^ ^28^ ^]^ In the present system, a similar effect likely occurred for **I@Ag54** to exhibit photoluminescence with high Φ_PL_.

To confirm the presence of an internal heavy‐atom effect, the PL decay curves of **X@Ag54** in deuterated toluene were evaluated using the time‐correlated single‐photon counting method (Figure 6B). The obtained decay curves were fitted with multiple exponential decays to determine the average photoluminescence lifetimes (*τ*_PL_) of **S@Ag54** and **I@Ag54**, which were 106.2 and 652.9 ns, respectively (Table 1; Table S10, Supporting Information). From this fitting, the radiative rate constants (*k*_r_) of **S@Ag54** and **I@Ag54** were estimated to be 1.9 × 10^3^ and 4.8 × 10^4^ s^−1^, respectively (Table 1). Therefore, when X changed from S^2−^ to I^−^, *k*_r_ increased by ≈25 times. This trend is similar to the enhancement of phosphorescent parameters through the heavy atom effect in conventional fluorescent dyes. Therefore, it can be interpreted that replacing S^2−^ with heavier I^−^ as X in **X@Ag54** accelerated ISC via the heavy atom effect,^[^ ^29^ ^]^ which increases the population of the bright triplet state and *k*_r_, thereby improving the Φ_PL_ of **X@Ag54**.

The non‐radiative rate constant (*k*_nr_) of **I@Ag54** was ≈1/6 of that of **S@Ag54** (Table 1). Because I^−^ has a larger ionic radius than S^2−^, it fills a greater volume of the cavity than S^2−^, and thus **I@Ag54** seems to have a more rigid framework than **S@Ag54**. In addition, the bond enthalpies of Ag─I and Ag─S bonds are 234 and 217 kJ mol^−1^, respectively, indicating that the Ag─I bond has a slightly higher bond energy than the Ag—S bond.^[^ ^30^ ^]^ These factors seems to accelerate the vibrational relaxation of **I@Ag54** compared with that of **S@Ag54**, thereby reducing the non‐radiative rate constant of **I@Ag54** to ≈1/6 of that of **S@Ag54**.

### 2.8 Optical Absorption

Finally, we discuss about the optical absorption of **S@Ag54** and **I@Ag54** (Figure 6C). Both spectra exhibited shoulder‐like absorptions ≈400 and 550 nm, and their overall optical absorption spectra were quite similar to each other. However, upon closer inspection, the spectrum of **I@Ag54** contained a more distinct shoulder‐like peak at ≈500 nm compared with the spectrum of **S@Ag54**. The heavy atom effect increases the oscillator strength of the spin‐forbidden transition from the ground state to the triplet excited state (S_0_→T_1_), leading to the S─T absorption peak in the absorption spectrum.^[^ ^31^ ^]^ In fact, in the optical absorption spectrum of some ligand‐protected metal clusters, the absorption peak attributable to S─T transition appears with a relatively high oscillator strength at a slightly longer wavelength than the peak attributed to S_0_→S*_n_* transition.^[^ ^11^ ^]^ Therefore, we predict that the shoulder peak at ≈500 nm in the optical absorption spectrum of **X@Ag54** is derived from S─T absorption, which is promoted by the heavy atom effect (Figure 6D). The assignment of these optical absorption peaks is expected to be clarified through DFT calculations^[^ ^32^ ^]^ in future studies.

## 3 Conclusion

In this study, we successfully synthesized a pair of **X@Ag54** clusters (X = S or I) protected by two types of ligands, thiolate and sulfonate, by adding Ag^+^ and a catalytic amount of Cu^2+^ to the reaction system. No significant difference was observed between the two **X@Ag54** clusters (X = S or I) in terms of geometric structure and stability against degradation. However, notable differences were observed in their PL and optical absorption. We concluded that the differences in their optical properties are derived from the heavy atom effect. This effect enhances the oscillator strength of transitions from the spin‐forbidden excited singlet state to the triplet excited state, as well as transitions from the ground singlet state to the triplet excited state. This study clearly demonstrated that incorporating heavy atoms into Ag─S clusters significantly enhances their phosphorescence quantum yield, similar to organic fluorescent dyes. These findings are expected to provide clear design guidelines for developing metal clusters as room‐temperature phosphorescent materials and triplet sensitizers.

## Conflict of Interest

The authors declare no conflict of interest.

## Supporting information



Supporting Information

Supporting Information

## Data Availability

The data that support the findings of this study are available from the corresponding author upon reasonable request.
