# Supplementary material for: Enhancement of Photoluminescence Quantum Yield of Silver Clusters by Heavy Atom Effect
Source: Small. 2025 Mar 3;21(25):2500700. doi: 10.1002/smll.202500700 (PMC12199116; doi:10.1002/smll.202500700)
Supplement: Supplementary file 1 — Supporting Information [file SMLL-21-2500700-s001.pdf]

NANO · MICRO
small

Supporting Information

for *Small*, DOI 10.1002/smll.202500700

Enhancement of Photoluminescence Quantum Yield of Silver Clusters by Heavy Atom Effect

Aoi Akiyama, Sakiat Hossain, Yoshiki Niihori, Kazutaka Oiwa, Jayoti Roy, Tokuhisa Kawawaki, Thalappil Pradeep and Yuichi Negishi**

Supporting Information

Enhancement of Photoluminescence Quantum Yield of Silver Clusters by Heavy Atom Effect

Aoi Akiyama, Sakiat Hossain, Yoshiki Niihori, Kazutaka Oiwa, Jayoti Roy, Tokuhisa Kawawaki, Thalappil Pradeep, and Yuichi Negishi**

Table of Contents

S1. Experimental································· Page 3

S2. Calculation································· Page 6

S3. Discussion································· Page 7

S4. Additional Scheme································· Page 8

S5. Additional Tables································· Page 9

S6. Additional Figures································· Page 15

S7. References································· Page 25

S1 Experimental

S1.1 Chemicals

All chemicals were obtained commercially and were used without further purification. Silver(I) trifluoroacetate (Ag(TFA)), copper(II) nitrate trihydrate ($Cu(NO_3)_2{\cdot}3H_2O$) were from FUJIFILM Wako Pure Chemical Co. *tert*-Butyl mercaptan (^{t}BuSH), tetrabutylammonium iodide (TBAI), coumarin 153 were from Tokyo Chemical Industry Co. Acetonitrile, acetone, toluene, and chloroform-d_1 ($CDCl_3$) were from Kanto Chemical Co., Inc.

S1.2 Detail of Synthesis

The glass tube used for synthesis and crystal growth was made by MARUEMU CO., INC. (part number: STC-4), with an outer diameter of 10 mm and a height of 75 mm **(Figure S1A)**. The cap made of polyethylene at the top has a single hole with a diameter of 0.8 mm **(Figure S1B)**. The raw material solution was poured into the glass tube and capped with the holed cap. At this time, the solution was confirmed to be light blue in color and free of the smell of ^{t}BuSH. A white LED (PENDY.LD, UW-SP19) was placed 13 cm above the top of the glass tube and the LED light was continuously irradiated until orange crystals were formed **(Figure S1C)**. When a similar synthesis experiment was conducted in a closed system, sulfonate formation and cluster formation did not occur due to the lack of oxygen inflow.

After approximately two weeks, the orange crystals were collected and dissolved in chloroform. To this solution, *n*-hexane was slowly poured to prepare a single crystal for X-ray diffraction **(Figure S2B)**.

S1.3 Single Crystal X-ray Diffraction and Crystallographic Data

The single crystal X-ray diffraction (SC-XRD) data were collected using a Bruker D8 QUEST diffractometer equipped with a fine-focus sealed X-ray tube producing multi-layer mirror-monochromated Mo-Kα radiation (λ = 0.71073 Å). The crystal was kept at −173(2) °C during data collection. The structure was solved by APEX4 program. Final refinement was performed by SHELXL-2018/3 using the Olex 2 platform. The crystallographic data were summarized in **Table S1**.

S1.4 X-ray Photoelectron Spectroscopy

X-ray photoelectron spectroscopy (XPS) was performed using JEOL's JPS-9010MC. The sample was placed on an indium plate, irradiated with Mg-Kα (1253.6 eV) radiation under reduced pressure of ~2×10^{-8} Torr, and the photoelectron spectra were measured. After measurement, the spectra were corrected using reference values for In $3d_{5/2}$ (443.8 eV) [1]. The Sherwood functions were used for the fitting curves **(Figure 3, S6, S7 and Table S4)**.

S1.5 Fourier Transform Infrared Absorption Spectroscopy

Fourier transform infrared (FT-IR) absorption spectra were recorded using a JASCO FT/IR-4600 spectrometer. X@Ag54 sample was dissolved in chloroform and deposited onto a diamond prism, followed by solvent evaporation. The spectrum of the prepared sample was then measured in the range of 400 to 4000 cm^{-1}. The results of IR spectra are depicted in **Figure S8**.

S1.6 Electrospray Ionization Mass Spectrometry

For the infusion analysis by electrospray ionization mass spectrometry (ESI-MS), we used MicroToF, Bruker and otofControl system. **X@Ag54** was dissolved in chloroform containing methanol and electrospray ionization mass spectrometry was attempted in positive ion mode. The spray voltage was set to 6000 V, the desolvation temperature to 200 °C, and the flow rate to 200 µL/h. However, multiple peaks were observed in both X@Ag54 clusters, and the nondestructive peaks attributed to $X@Ag_{54}S_{20}(S^tBu)_{20}(SO_3{}^tBu)_m$ (X, m) = (S, 12), (I, 11)

obtained by single crystal X-ray diffraction and ^{1}H NMR were not observed **(Figure S3 and Table S2, S3)**.

S1.7 Thermal Gravitational Analysis

Thermogravimetric (TG) curves and differential thermal analysis (DTA) of the samples were measured using Bruker, TG-DTA 2010SA. Before measurement, the samples were heated at 60 °C and dried under vacuum condition for 10 hours. Measurements were performed under nitrogen flow conditions at 50 mL/min and a temperature increase rate of 7 °C/min from room temperature to 800°C.

S1.8 Collision Induced Dissociation and Their Analysis

ESI-MS studies were performed using a Waters Synapt G2-Si high-definition mass spectrometer (HDMS). All mass spectrometric measurements were performed in the positive ion mode. For ESI-MS studies, **X@Ag54** nanocluster was dissolved in a solvent mixture of chloroform : methanol (1 : 1). During ESI, the capillary voltage, sampling cone, and source offset were kept at 3.2 kV, 10V, and 0 V, respectively, to obtain a well resolved mass spectrum of the **X@Ag54** nanocluster. The source temperature and desolvation temperature were maintained at 100 °C and 150 °C, respectively. Desolvation gas flow and cone gas flow were set to 400 and 10 L h^{-1}, respectively. In the Synapt G2-Si instrument, the mass-selected ions pass through trap, ion mobility and transfer cells before entering the time-of-flight analyzer. The collision induced dissociation (CID) of isolated ion can be performed at trap and transfer cells after isolating the ion using quadrupole mass filter. In our experiments, we carried out CID in both trap and transfer cells, while the IMS cell was kept off. No extra voltages were applied in the IMS cell to avoid additional fragmentation of the ions. The trap and transfer cells were filled with inert Ar gas. In trap, the collision energy (CE) was varied between 0 and 200 V. Further increase of CE was attained by increasing the voltages in the transfer cells. As the IMS cell did not contribute any effect on the applied collision voltages, the total CE is considered as the sum of the trap and transfer CEs (in V).[2] The results are summarized in **Figure S13**, **S14** and **Table S7**, **S8**.

From the CID mass spectrum at each collision energy (E_{CE}), the survival yield (SY) of the parent ion was calculated using the following formula.

$$SY = \frac{I_{\mathrm{P}}}{\sum I_{\mathrm{F}} + I_{\mathrm{P}}} \qquad (\textbf{Equation S1})$$

E_{CE} was converted to center-of-mass kinetic energy using the following equations, where m_{Ar} and m_{P} are the mass number of the colliding gas (Ar) and parent ion, respectively.

$$E_{\mathrm{COM}} = \frac{m_{\mathrm{Ar}}}{m_{\mathrm{Ar}} + m_{\mathrm{P}}} E_{\mathrm{CE}}. \qquad (\textbf{Equation S2})$$

The CE dependence of SY of the parent ion was fitted by the following sigmoid function **(Figure 5B)**.

$$SY = \frac{1}{1 + \exp\left(\frac{E_{\mathrm{COM}} - E_{50}}{c}\right)}. \qquad (\textbf{Equation S3})$$

, where E_{COM} is the collision energy, c is the curvature in the sigmoid curve, and E_{50} is the collision energy when SY is 50% **(Table S9)**.

S1.9 Kinetic Analysis of Decomposition of X@Ag54 in Solution

X@Ag54 was dissolved in toluene and the time evolution of absorbance was monitored **(Figure S11)**. To perform a reaction rate analysis, a decay process according to a first-order rate equation was considered. However, **X@Ag54** was stable and did not decay significantly after 20 hours. Therefore, we attempted to analyze the kinetics near t = 0 by Taylor

expansion of the analytical solution of the first-order differential rate equation. The analytical solution obtained from the first-order differential velocity equation is expressed as follows.

$$[\mathrm{M}]_t = [\mathrm{M}]_0 \exp(-kt). \qquad \textbf{(Equation S4)}$$

, where M represents **X@Ag54** and $[\mathrm{M}]_t$ is the concentration of M at time *t*. *k* is the rate constants for the assumed first-order decays. If we perform a Taylor expansion of this analytical solution near *t* = 0 and omit the terms after the second order, we obtain following relation.

$$[\mathrm{M}]_t = [\mathrm{M}]_0 \sum_{i=0}^{\infty} \frac{(-kt)^i}{i!} \approx [\mathrm{M}]_0(1 - kt). \qquad \textbf{(Equation S5)}$$

Since concentration is proportional to absorbance, the above equation can be rewritten as follows.

$$A_t \approx -A_0 kt + A_0. \qquad \textbf{(Equation S6)}$$

The unknown parameters A_0 and *k* were estimated using the least squares method, with the above equation serving as the fitting function for **Figure S11**. The fitting results were described in **Table S5**.

S1.10 Photoluminescence Spectroscopy and Their Analysis

First, **X@Ag54** was dissolved in toluene and the solution was poured into quartz cuvette with optical length of 1.0 cm. Oxygen molecules in the solution were removed from the solution by bubbling argon containing saturated toluene vapor. The emission spectra of sample ($I_X(\lambda_{em})$) were measured under deoxygenated conditions by irradiation of 405 nm excitation light using JASCO's FP-6300 and the absorbance $A(\lambda_{ex})$ at the excitation wavelength was measured by JASCO's V-770. The emission spectra (F_{norm}) **(Figure 6C)** normalized by absorptance was obtained using the following equation:

$$F_{\mathrm{norm}}(\lambda_{\mathrm{em}}) = \frac{I_{\mathrm{X}}(\lambda_{\mathrm{em}})}{1 - 10^{-A(\lambda_{\mathrm{ex}})}}. \qquad \textbf{(Equation S7)}$$

The photoluminescence quantum yield (PLQY, Φ_{PL}) of **X@Ag54** was determined by the relative method[3] using coumarin 153 (Φ_{std} = 0.544 in deaerated ethanol)[4] as a standard sample. The obtained values were substituted into the following formula to evaluate the relative spectral quantum yield of the unknown sample X.

$$\Phi_{\mathrm{PL,X}} = \frac{1 - 10^{-A_{\mathrm{std}}(\lambda_{\mathrm{ex}})}}{1 - 10^{-A_{\mathrm{X}}(\lambda_{\mathrm{ex}})}} \cdot \frac{\int I_{\mathrm{X}}(\lambda_{\mathrm{em}})\mathrm{d}\lambda_{\mathrm{em}}}{\int I_{\mathrm{std}}(\lambda_{\mathrm{em}})\mathrm{d}\lambda_{\mathrm{em}}} \cdot \left(\frac{n_{\mathrm{X}}}{n_{\mathrm{std}}}\right)^2 \cdot \Phi_{\mathrm{std}}. \qquad \text{(Equation S8)}$$

, where λ_{ex} and λ_{em} are the excitation and emission wavelengths, respectively, *n* is the refractive index of the solvent ($n_{toluene}$ = 1.496, $n_{ethanol}$ = 1.361). The integration of PL spectrum represents the area of PL spectrum.

S1.11 Photoluminescence Lifetime

Time-correlated single photon counting measurement was conducted by using UNISOKU's Pico-TAS. **X@Ag54** was dissolved in toluene and the solution was degassed by bubbling with argon containing saturated toluene vapor. A 405 nm pulse laser as the excitation source was used to evaluate the PL decay curve of **X@Ag54 (Figure 6B)**. The PL decay curve $I_{PL}(t)$ was fitted by the least-squares method using the following multiple exponential function:

$$I_{\mathrm{PL}}(t) = \sum_{i=1}^{3} A_i \exp\left(-\frac{t}{\tau_i}\right) + B. \qquad \text{(Equation S9)}$$

, where *A* is the amplitude (pre-exponential factor), *B* is the background, and *τ* is the emission lifetime. The fractional amplitude *α* and fractional population *f* were calculated as follows:

$$\alpha_i = \frac{A_i}{\sum_{j=1}^{3} A_j}, \qquad f_i = \frac{\alpha_i \tau_i}{\sum_{j=1}^{3} \alpha_j \tau_j}. \qquad \text{(Equation S10)}$$

The lifetime of **X@Ag54** was evaluated as the average PL lifetime based on the number of emitted photons using the following formula:

$$\tau_{\mathrm{PL}} = \sum_{i=1}^{3} f_i \tau_j \qquad (\textbf{Equation S11})$$

The fitting results were shown in **Table S10**.

S1.12 Radiative and Non-radiative Rate Constant

The radiative and non-radiative rate constant (k_{r} and k_{nr}, respectively) were estimated by the following equations.

$$k_{\mathrm{r}} = \frac{\Phi_{\mathrm{PL}}}{\tau_{\mathrm{PL}}}, \qquad k_{\mathrm{nr}} = \frac{1-\Phi_{\mathrm{PL}}}{\tau_{\mathrm{PL}}}. \qquad (\text{Equation S12})$$

The radiative lifetime (τ_{r}) is a reciprocal of k_{r} as follows.

$$\tau_{\mathrm{r}} = \frac{1}{k_{\mathrm{r}}}. \qquad (\text{Equation S13})$$

S1.13 Electron Spin Resonance Spectroscopy

We conducted X-band electron spin resonance (ESR) measurements to monitor the oxidation of thiols to sulfonates catalyzed by Cu and the promotion of thiol oxidation by the addition of Ag. $Cu(NO_3)_2{\cdot}3H_2O$ 8.0 mg was dissolved in 3 mL of an acetone/acetonitrile (50v%/50v%) mixture, followed by the addition of 3.5 mg of Ag(TFA) or none. This solution was transferred to a reaction tube **(Figure S1A)**, where 11 μL of ^{t}BuSH was added to initiate the reaction. At designated reaction times, aliquots of the reaction solution were collected into ESR tubes, and ESR spectra were recorded at room temperature. An EMX-nano spectrometer (Bruker, Boston, MA, USA) was used for the ESR measurements.

S2. Calculation

Density functional theory (DFT) calculations with the program Gaussian 16 (ES64L-G16, RevB.01) [5] were performed to investigate the dissociation energies at Ag−O and AgS−O_3S^tBu of the **X@Ag54** surface. B3LYP was used for the functional, with LanL2DZ (for Ag) [6] and 6-31G(d,p) (for H, C, O, and S) [7] basis sets. For Ag atoms, effective core potential was adapted. To simplify the system, only the Ag−O_3S^tBu and AgS−^{t}Bu binding units were optimized instead of the full structure of X@Ag54. In this system, we didn't consider basis set superposition error (BSSE), since these Ag−O and S−C bonds are strong covalent bonds. The Ag−O and S−C binding energies in Ag−O_3S^tBu and AgS−^{t}Bu, respectively, were then calculated by subtracting the energy of the optimized structure of each fragment from the total electronic energy of each unit. The total electronic energies of each unit and fragment are summarized in **Table S6**. As the results, we obtained following dissociation energy ΔE.

$$\mathrm{Ag}-\mathrm{O}_3S^{\mathrm{t}}\mathrm{Bu} \;\rightarrow\; \mathrm{Ag}\cdot \;+\; \cdot\,\mathrm{O}_3S^{\mathrm{t}}\mathrm{Bu}, \qquad \Delta E = 54.21\ \mathrm{kcal\ mol^{-1}}$$

$$\mathrm{AgS}-{}^{\mathrm{t}}\mathrm{Bu} \;\rightarrow\; \mathrm{AgS}\cdot \;+\; \cdot\,{}^{\mathrm{t}}\mathrm{Bu}, \qquad \Delta E = 57.38\ \mathrm{kcal\ mol^{-1}}$$

S3. Discussion

S3.1 Mixed-valence state

Here we consider the valence state of silver atoms in X@Ag54. In the case of **X@Ag54** ($X@Ag_{54}S_{20}(S^tBu)_{20}(SO_3{}^tBu)_m$), the contribution of the number of donor electrons from the ligand, sulfide, and X anion to the molecule can be calculated as $x + m + 60$ {(x, m) = (2, 12) and (1, 11) for X = S and I, respectively}, since X, S, S^tBu, and $SO_3{}^tBu$ can donate x, 2, 1, and 1 electrons, respectively.

Let y be the average oxidation number per Ag atom; then, the following equation holds: $54y = x + m + 60$. Solving this equation for y, the averaged oxidation number of Ag atom in S@Ag54 and I@Ag54 are calculated to be $+1.37$ and $+1.33$, respectively.

On the other hand, if the number of Ag^{II} atoms among the 54 Ag atoms is n, the number of Ag^{I} atoms can be $54 - n$, so the number of accepted electrons can be $2n + (54 - n)$ electrons. When **X@Ag54** has neutral charge and is 0-electron system, these numbers of donated and accepted electrons balance out and we obtain the equation $2n + (54 - n) = x + m + 60$. Solving the equation for n, the mixed-valence states of Ag in **S@Ag54** and **I@Ag54** can be **$S@Ag^{I}_{34}Ag^{II}_{20}$** and **$I@Ag^{I}_{36}Ag^{II}_{18}$**, respectively.

S3.2 Heavy Atom Effect

The ISC rate constant k_{ISC} can be expressed as Fermi's golden rule by adapting first-order perturbation theory to the Schrödinger equation of time evolution as follows. [8]

$$k_{\mathrm{ISC}} \propto \frac{\left\langle \Psi_{\mathrm{T}_m} \middle| \hat{H}_{\mathrm{SOC}} \middle| \Psi_{\mathrm{S}_n} \right\rangle^2}{\Delta E_{\mathrm{ST}}{}^2}. \qquad \textbf{(Equation S14)}$$

, where Ψ is the wave function and k_{ISC} is proportional to the square of the matrix elements from the singlet (S_n) and triplet (T_m) wave functions and the spin-orbit coupling Hamiltonian (H_{SOC}). ΔE_{ST} is energy difference between singlet and triplet state. H_{SOC} is also expressed as the inner product of the proportionality constant ζ, the orbital angular momentum operator $\hat{\boldsymbol{l}}$, and the spin angular momentum operator $\hat{\boldsymbol{s}}$, as follows.

$$\hat{H}_{\mathrm{SOC}} = \zeta \sum_i \hat{l}_i \cdot \hat{s}_i. \qquad \textbf{(Equation S15)}$$

Based on the experimental results, the absorption and emission spectra of **S@Ag54** and **I@Ag54** did not change significantly, so the ΔE_{ST} of **Equation S14** should not change significantly. If the electronic states involved in ISC were unchanged between **S@Ag54** and **I@Ag54**, the rate of change of k_{ISC} from S ($\zeta = 2.88 \times 10^2$ cm^{-1}) to I ($\zeta = 4.96 \times 10^3$ cm^{-1})[9] would be 297 times the ratio of ζ squared, but since this value is one order of magnitude different from the ratio of k_r (25 times), the inclusion of S or I would cause a change in the electronic states involved in ISC, and consequently the matrix element values may also change. In addition to the structure optimization of the ground state of **X@Ag54** and the vertical transition calculation, the structure optimization of the excited singlet and triplet states is also necessary to clarify these excitation and relaxation processes in detail, but at present no appropriate theory-based calculation method has been established.

S4. Additional Scheme

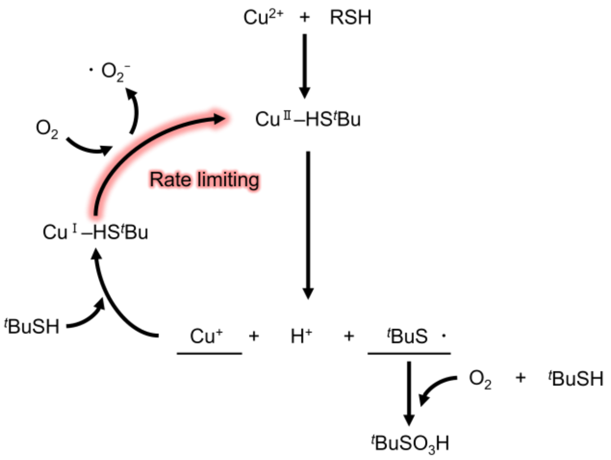

Scheme S1. Oxidation of thiol by Cu catalyst.

S5. Additional Tables

Table S1. Crystal data and structure refinement

	S@Ag54	I@Ag54
CCDC No.	2407225	2407226
Empirical formula	$C_{128}H_{288}Ag_{54}O_{36}S_{53}$	$C_{128}H_{282}Ag_{54}IO_{36}S_{52}$
Formula weight	9927.75	10016.39
Temperature / K	100.15	100.15
Crystal system	cubic	cubic
Space group	*Fm*−3	*Pa*−3
a / Å	32.1653(17)	29.8732(8)
b / Å	32.1653(17)	29.8732(8)
c / Å	32.1653(17)	29.8732(8)
α / °	90	90
β / °	90	90
γ / °	90	90
Volume / $Å^3$	33278(5)	26659(2)
Z	4	4
ρ_{calc} / $g{\cdot}cm^{-3}$	1.982	2.496
μ / mm^{-1}	3.462	4.430
F(000)	18920.0	19044.0
Crystal size / mm^3	0.2 × 0.2 × 0.2	0.2 × 0.2 × 0.2
Radiation (wavelength)	Mo Kα (λ = 0.71073)	Mo Kα (λ = 0.71073)
2Θ range for data collection / °	4.2 to 41.64	3.34 to 35.984
Index ranges	-28 ≤ *h* ≤ 32, -23 ≤ *k* ≤ 32, -32 ≤ *l* ≤ 30	-25 ≤ *h* ≤ 25, -25 ≤ *k* ≤ 19, -20 ≤ *l* ≤ 25
Reflections collected	12516	136245
Independent reflections	1564 [R_{int} = 0.0949, R_{sigma} = 0.0427]	7824 [R_{int} = 0.0953, R_{sigma} = 0.0180]
Data/restraints/parameters	1564/181/139	7824/223/393
Goodness-of-fit on F_2	1.026	1.073
Final R indexes [*I* >= 2σ (*I*)]	R_1 = 0.0788, $_wR_2$ = 0.1785	R_1 = 0.1058, $_wR_2$ = 0.5874
Final *R* indexes [all data]	R_1 = 0.1049, $_wR_2$ = 0.2002	R_1 = 0.1053, $_wR_2$ = 0.2138
Largest diff. peak/hole / e $Å^{-3}$	2.42/-2.13	6.41/-3.43

Table S2. Assignment of peaks in ESI mass spectrum of **S@Ag54 (Figure S3A)**

Peak	Assignment [a]	Exp. *m/z* [b]	Cal. *m/z* [b]
a	S@Ag54 – S^tBu + $SO_3{}^tBu$ + AgS	5057.7	5058.2
b	S@Ag54 + $SO_3{}^tBu$	5032.2	5032.2
c	S@Ag54 + AgS + ^tBu – Ag	5008.4	5008.2
d	S@Ag54 + AgS + 2 ^tBu – 2 Ag	4983.0	4983.3

a) S@Ag54 = $SAg_{54}S_{20}(S^tBu)_{20}(SO_3{}^tBu)_{12}$; b) *m/z* giving maximum ion intensity.

Table S3. Assignment of peaks in ESI mass spectrum of **I@Ag54** (**Figure S3B**)

Peak	Assignment [a]	Exp. *m/z* [b]	Cal. *m/z* [b]
a	I@Ag54 + 2 AgS	5151.3	5151.1
b	I@Ag54 + 2 AgS + S^tBu – $SO_3{}^tBu$	5127.5	5127.1
c	I@Ag54 + 2 AgS + S^tBu – $SO_3{}^tBu$ + ^tBu – Ag	5102.0	5102.2
d	I@Ag54 + 2 AgS + 2 S^tBu – 2 $SO_3{}^tBu$ + ^tBu – Ag	5077.9	5078.2

a) I@Ag54 = $IAg_{54}S_{20}(S^tBu)_{20}(SO_3{}^tBu)_{11}$; b) *m/z* giving maximum ion intensity.

Table S4. Fitting result of X-ray photoelectron spectra of **X@Ag54** (**Figure 3**)

Sample	Assignment	Energy / eV	FWHM / eV [a]	Ratio [b]
**S@Ag54**	Ag $3d_{5/2}$	367.404	0.501096	23.7850
		368.001	0.854979	43.7911
	Ag $3d_{3/2}$	373.418	0.430232	12.5033
		374.052	0.786515	19.9206
	S $2p_{3/2}$ (Ag_2S or AgS^tBu)	161.998	1.3118	78.3707
	S 2p ($SO_3{}^tBu$)	167.575	1.14173	21.6293
**I@Ag54**	Ag $3d_{5/2}$	367.223	0.544069	22.5915
		367.734	0.643328	47.1520
	Ag $3d_{3/2}$	373.201	0.456266	11.5903
		373.880	0.603143	18.6661
	S 2p (Ag_2S or AgS^tBu)	161.675	1.221	65.7059
	S 2p ($SO_3{}^tBu$)	167.248	1.03238	34.2941
	I $3d_{5/2}$	618.586	0.879174	72.5754
	I $3d_{3/2}$	629.974	0.720445	27.4246

a) Full width of half maxima; b) Area ratio in one element.

Table S5. First-order kinetic analysis of decomposition of **X@Ag54** in toluene (60 °C) (**Figure S11**)

Sample	A_0 [a]	k / 10^{-3} h^{-1} [b]	$\tau_{1/2}$ / h (/ d) [c]
**S@Ag54**	0.3689	2.86	242 (10.1)
**I@Ag54**	0.2438	3.75	185 (7.70)

a) Initial absorbance; b) First-order rate constant; c) Half-life.

Table S6. Total electron energy (E) of optimized binding units and fragments[a]

Binding unit (E / Hartree)	Fragment 1 (E / Hartree)	Fragment 2 (E / Hartree)
Ag−O_3S^tBu (−927.4464)	Ag· (−145.7587)	·O_3S^tBu (−781.6013)
AgS−^{t}Bu (−701.8360)	AgS· (−543.9336)	·^{t}Bu (−157.8110)

a) B3LYP/LanL2DZ(Ag),6-31G(d,p)(H,C,O,S).

Table S7. Assignment of peaks in CID mass spectra of **S@Ag54** (**Figure S13**)

Assignment [a]		Exp. *m/z* [b]	Cal. *m/z* [b]
{S@Ag54 − S^{t}Bu + AgS + SO_3^{t}Bu}		5057.7	5058.2
{S@Ag54 + SO_3^{t}Bu}		5034.2	5032.2
{S@Ag54 − S^{t}Bu + AgS + SO_3^{t}Bu}	− 1 ^{t}Bu − 1 SO_3^{t}Bu	4960.7	4961.1
{S@Ag54 + SO_3^{t}Bu}	− 1 ^{t}Bu − 1 SO_3^{t}Bu	4937.1	4935.2
{S@Ag54 − S^{t}Bu + AgS + SO_3^{t}Bu}	− 2 ^{t}Bu − 2 SO_3^{t}Bu	4863.7	4863.1
{S@Ag54 + SO_3^{t}Bu}	− 2 ^{t}Bu − 2 SO_3^{t}Bu	4840.2	4838.1
{S@Ag54 − S^{t}Bu + AgS + SO_3^{t}Bu}	− 3 ^{t}Bu − 3 SO_3^{t}Bu	4766.6	4766.0
{S@Ag54 + SO_3^{t}Bu}	− 3 ^{t}Bu − 3 SO_3^{t}Bu	4743.0	4741.1
{S@Ag54 − S^{t}Bu + AgS + SO_3^{t}Bu}	− 4 ^{t}Bu − 4 SO_3^{t}Bu	4669.5	4669.0
{S@Ag54 + SO_3^{t}Bu}	− 4 ^{t}Bu − 4 SO_3^{t}Bu	4645.3	4644.0
{S@Ag54 − S^{t}Bu + AgS + SO_3^{t}Bu}	− 5 ^{t}Bu − 5 SO_3^{t}Bu	4572.4	4571.9
{S@Ag54 + SO_3^{t}Bu}	− 5 ^{t}Bu − 5 SO_3^{t}Bu	4548.5	4547.0
{S@Ag54 − S^{t}Bu + AgS + SO_3^{t}Bu}	− 6 ^{t}Bu − 6 SO_3^{t}Bu	4475.5	4474.9
{S@Ag54 + SO_3^{t}Bu}	− 6 ^{t}Bu − 6 SO_3^{t}Bu	4451.5	4449.9
{S@Ag54 − S^{t}Bu + AgS + SO_3^{t}Bu}	− 7 ^{t}Bu − 7 SO_3^{t}Bu	4478.5	4377.8
{S@Ag54 + SO_3^{t}Bu}	− 7 ^{t}Bu − 7 SO_3^{t}Bu	4354.5	4352.9
{S@Ag54 − S^{t}Bu + AgS + SO_3^{t}Bu}	− 8 ^{t}Bu − 8 SO_3^{t}Bu	4281.5	4280.8
{S@Ag54 + SO_3^{t}Bu}	− 8 ^{t}Bu − 8 SO_3^{t}Bu	4257.5	4254.8
{S@Ag54 − S^{t}Bu + AgS + SO_3^{t}Bu}	− 9 ^{t}Bu − 9 SO_3^{t}Bu	4184.5	4183.7
{S@Ag54 + SO_3^{t}Bu}	− 9 ^{t}Bu − 9 SO_3^{t}Bu	4169.4	4157.8
{S@Ag54 − S^{t}Bu + AgS + SO_3^{t}Bu}	− 10 ^{t}Bu − 10 SO_3^{t}Bu	4087.3	4086.7
{S@Ag54 + SO_3^{t}Bu}	− 10 ^{t}Bu − 10 SO_3^{t}Bu	4062.3	4060.7
{S@Ag54 − S^{t}Bu + AgS + SO_3^{t}Bu}	− 11 ^{t}Bu − 11 SO_3^{t}Bu	3988.2	3989.6
{S@Ag54 + SO_3^{t}Bu}	− 11 ^{t}Bu − 11 SO_3^{t}Bu	3965.4	3963.7
{S@Ag54 − S^{t}Bu + AgS + SO_3^{t}Bu}	− 12 ^{t}Bu − 12 SO_3^{t}Bu	3892.6	3891.6
{S@Ag54 + SO_3^{t}Bu}	− 12 ^{t}Bu − 12 SO_3^{t}Bu	3867.2	3866.7

[a] S@Ag54 = $SAg_{54}S_{20}(S^tBu)_{20}(SO_3{}^tBu)_{12}$; [b] *m/z* giving maximum ion intensity.

Table S8. Assignment of peaks in CID mass spectra of **I@Ag54** (**Figure S14**)

Assignment [a]		Exp. *m/z* [b]	Cal. *m/z* [b]
{I@Ag54 + 2 AgS + $SO_3{}^tBu$}		5151.0	5151.1
{I@Ag54 + 2 AgS + $SO_3{}^tBu$}	− 1 ^tBu − 1 $SO_3{}^tBu$	5054.3	5054.0
{I@Ag54 + 2 AgS + $SO_3{}^tBu$}	− 2 ^tBu − 2 $SO_3{}^tBu$	4957.1	4957.0
{I@Ag54 + 2 AgS + $SO_3{}^tBu$}	− 3 ^tBu – 3 $SO_3{}^tBu$	4859.9	4859.9
{I@Ag54 + 2 AgS + $SO_3{}^tBu$}	− 4 ^tBu – 4 $SO_3{}^tBu$	4762.3	4762.9
{I@Ag54 + 2 AgS + $SO_3{}^tBu$}	− 5 ^tBu – 5 $SO_3{}^tBu$	4665.2	4665.8
{I@Ag54 + 2 AgS + $SO_3{}^tBu$}	− 6 ^tBu – 6 $SO_3{}^tBu$	4568.6	4568.8
{I@Ag54 + 2 AgS + $SO_3{}^tBu$}	− 7 ^tBu – 7 $SO_3{}^tBu$	4471.8	4470.7
{I@Ag54 + 2 AgS + $SO_3{}^tBu$}	− 8 ^tBu – 8 $SO_3{}^tBu$	4374.1	4373.7
{I@Ag54 + 2 AgS + $SO_3{}^tBu$}	− 9 ^tBu – 9 $SO_3{}^tBu$	4276.8	4276.6
{I@Ag54 + 2 AgS + $SO_3{}^tBu$}	− 10 ^tBu – 10 $SO_3{}^tBu$	4180.1	4179.6
{I@Ag54 + 2 AgS + $SO_3{}^tBu$}	− 11 ^tBu – 11 $SO_3{}^tBu$	4083.3	4082.5

[a] I@Ag54 = $IAg_{54}S_{20}(S^tBu)_{20}(SO_3{}^tBu)_{11}$; [b] *m/z* giving maximum ion intensity.

Table S9. Fitting results of survival yield obtained from CID profiles (**Figure 5B**)

Sample	E_{50} / E_{COM}	c / E_{COM}
**S@Ag54**	0.29370	0.01766
**I@Ag54**	0.30071	0.01647

[a] Coefficient of determination for the fitting.

Table S10. Fitting results of the PL decay curves of X@Ag54 in deaerated toluene (**Figure 6B**)

Sample	τ / ns	α [a]	f [b]	τ_{PL} / ns [c] (χ^2) [d]
**S@Ag54**	3.1	0.628	0.088	106.2 (1.0535)
	20.6	0.278	0.260	
	154.1	0.934	0.653	
**I@Ag54**	16.3	0.464	0.029	652.9 (1.0069)
	217.1	0.320	0.267	
	844.8	0.216	0.704	

[a] Fractional amplitude; [b] Fractional population; [c] Photon-number-based averaged lifetime; [d] Chi-square value for the fitting.

S6. Additional Figures

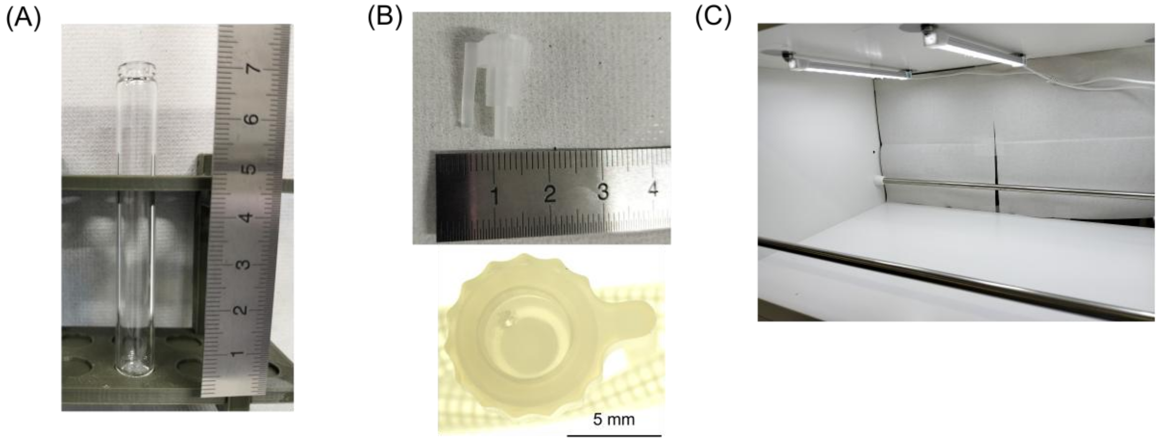

Figure S1. Photographs of (A) the tube for synthesis and crystal growth, (B) holed cap, and (C) light irradiation system.

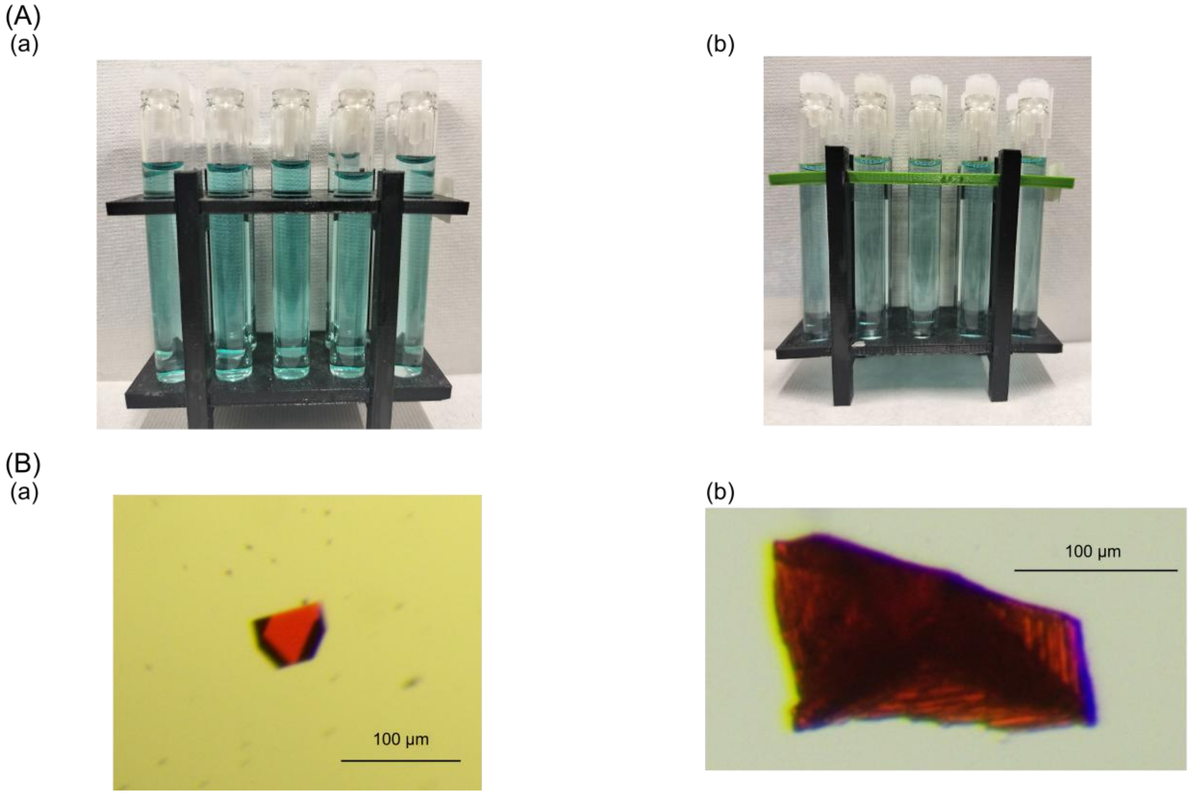

Figure S2. Photographs of (A) the reaction solution and (B) the single crystal for SC-XRD: (a) **S@Ag54** and (b) **I@Ag54**.

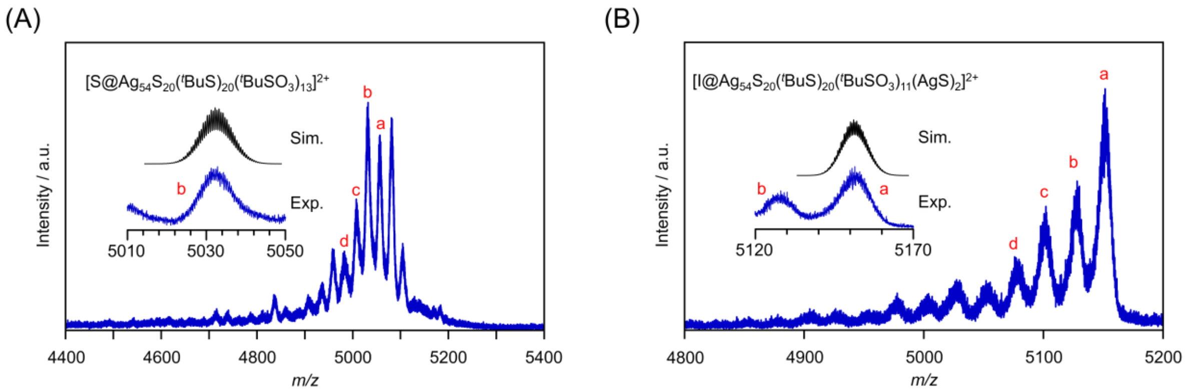

Figure S3. ESI-mass spectra of **X@Ag54** in the positive-ion mode. (A) **S@Ag54** and (B) **I@Ag54**. The assignment of each peak is summarized in **Table S2** (for **S@Ag54**) and **Table S3** (for **I@Ag54**).

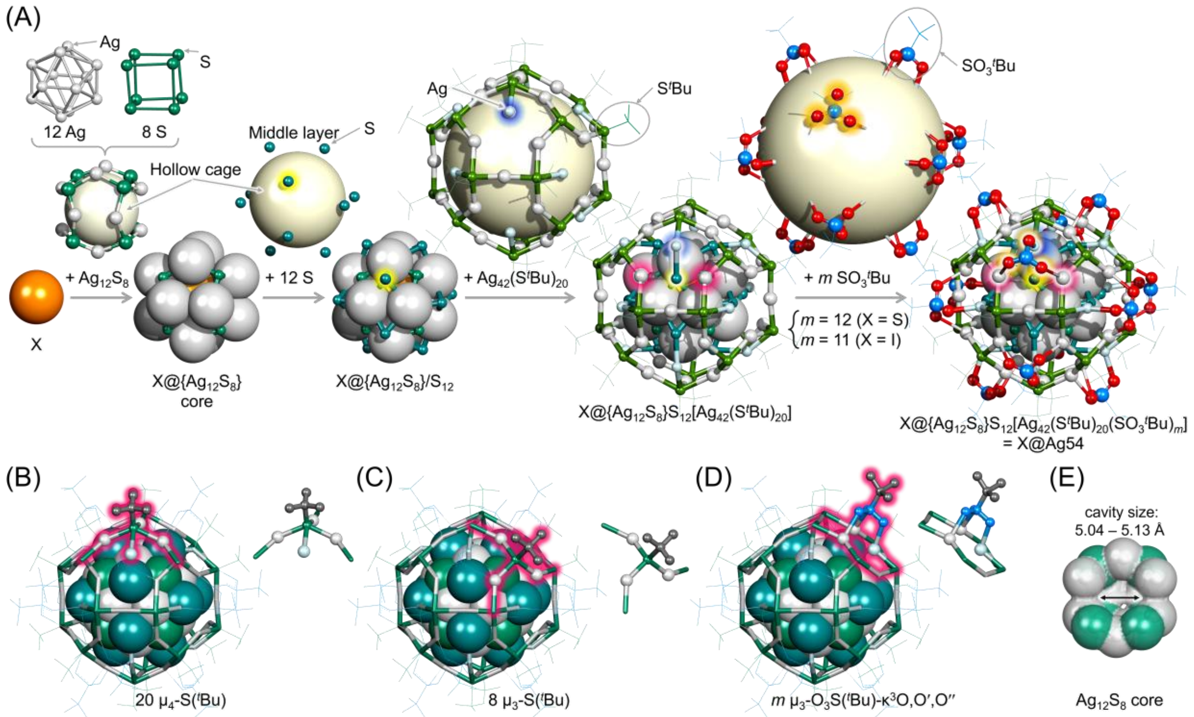

Figure S4. Details of the structure of **X@Ag54**. (A) Assembly of **X@Ag54**. (B) Structure of μ4-S(^{t}Bu). (C) Structure of μ3-S(^{t}Bu) (D) Structure of μ3-O3S(^{t}Bu)-κ^{3}O,O′,O″. (E) Cavity inside $Ag_{12}S_8$ core.

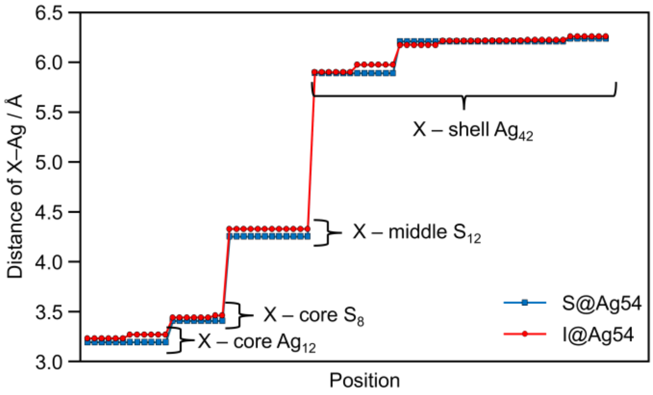

Figure S5. X−Y (Y = Ag, S) distance distribution in **X@Ag54**. The distances between the central atoms (S or I) and the nearest Ag atoms in **S@Ag54** and **I@Ag54** are 3.19 Å and 3.27 Å, respectively (**Figure S5**). Considering the ionic radius, **S@Ag54** can be considered to have no bond between S and the Ag atoms of core. However, this distance is shorter than the total value of van der Waals radius of S and Ag atoms. Therefore, it can be considered that a weak bond exists also in the core of **S@Ag54**.

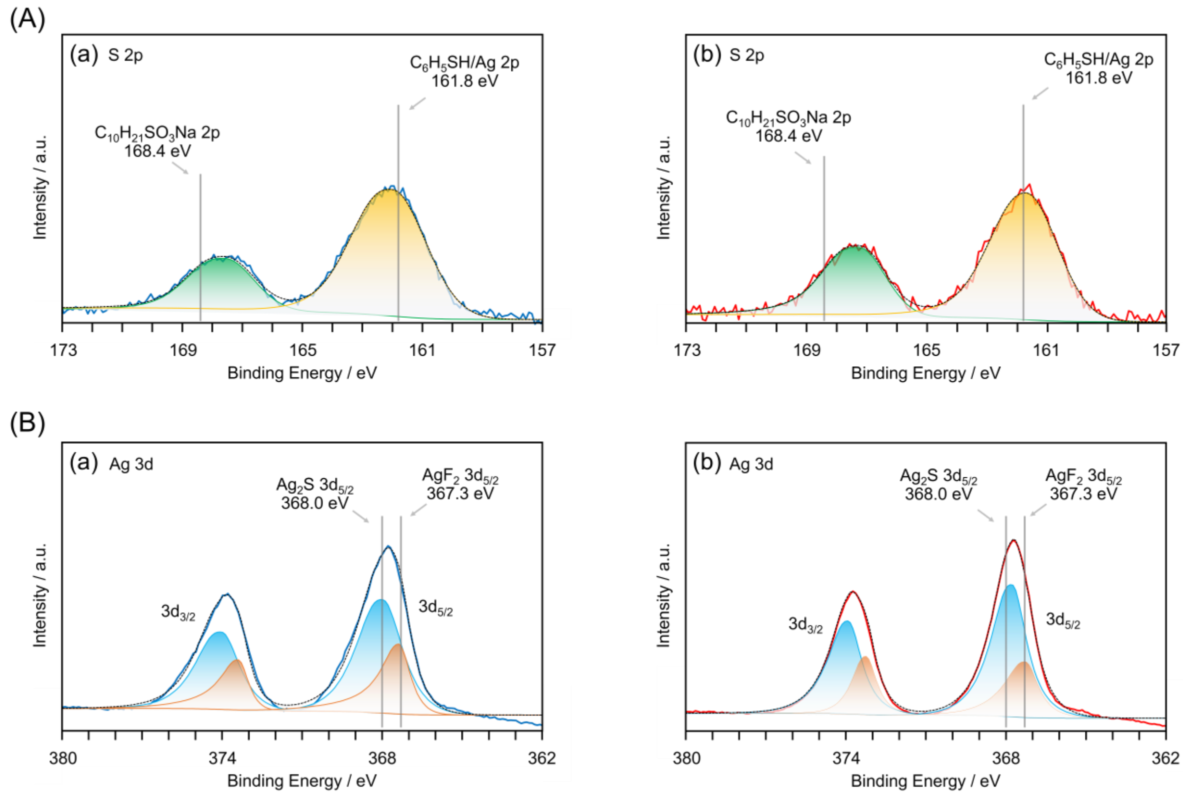

Figure S6. XPS spectra of (A) S 2p and (B) Ag 3d of (a) **S@Ag54** and (b) **I@Ag54**. The gray lines represent the values of binding energy from the references.[10] Figure (B) shows that the ratio of Ag^{I}:Ag^{II} is 63.7:36.3 and 65.8:34.2 for S@Ag54 and I@Ag54, respectively. As shown in Discussion of S3.1, when **X@Ag54** has neutral charge and is 0-electron system, the mixed-valence states of Ag in **S@Ag54** and **I@Ag54** can be estimated to be S@$Ag^{I}_{34}Ag^{II}_{20}$ and I@$Ag^{I}_{36}Ag^{II}_{18}$, respectively. In this composition, the ratio of Ag^{I} : Ag^{II} is 62.9 : 37.1 and 66.7 : 33.3 for S@Ag54 and I@Ag54, respectively. In this way, the results of Ag 3d XPS well reproduce the expected mixed-valence states of Ag for **S@Ag54** and **I@Ag54**.

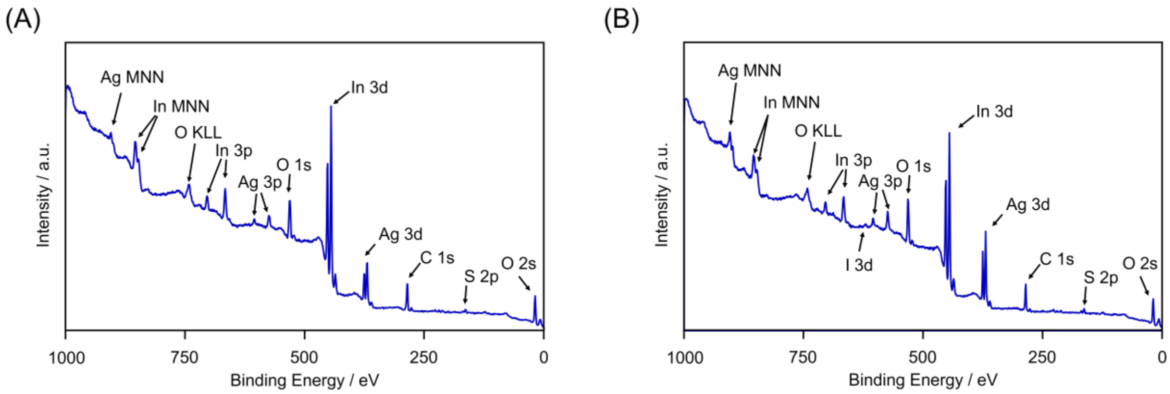

Figure S7. Wide-ranged X-ray photoelectron spectra of (A) **S@Ag54** and (B) **I@Ag54**. Indium was used as the substrate for calibration.

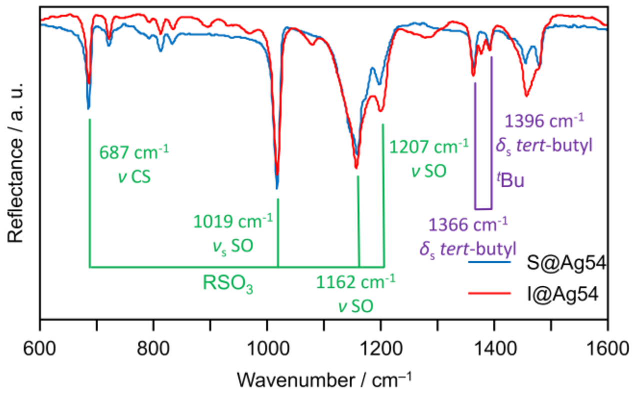

Figure S8. FT-IR absorption spectra of **X@Ag54**.

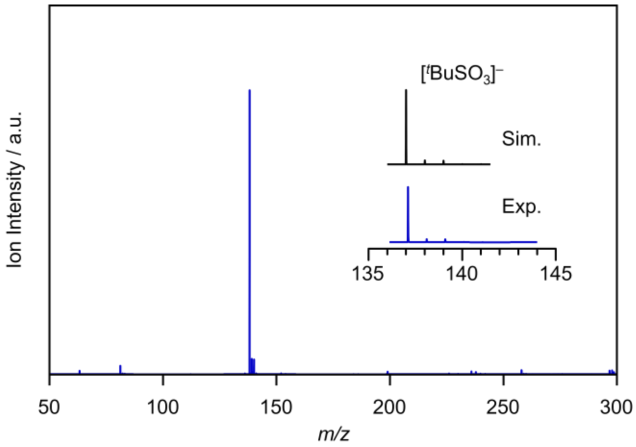

Figure S9. ESI-MS spectrum of ^{t}BuSO$_3^-$ produced from the corresponding thiol and oxygen molecule in the presence of $Cu(NO_3)_2$ catalyst. The inset shows the comparison of the isotope pattern between experimental and simulation spectra. The product was obtained by adding ^{t}BuSH dropwise into a mixed acetonitrile-acetone solution of $Cu(NO_3)_2 \cdot 3H_2O$, followed by standing for two days in the reaction vessel. The resulting white precipitate was dissolved in methanol for the measurement.

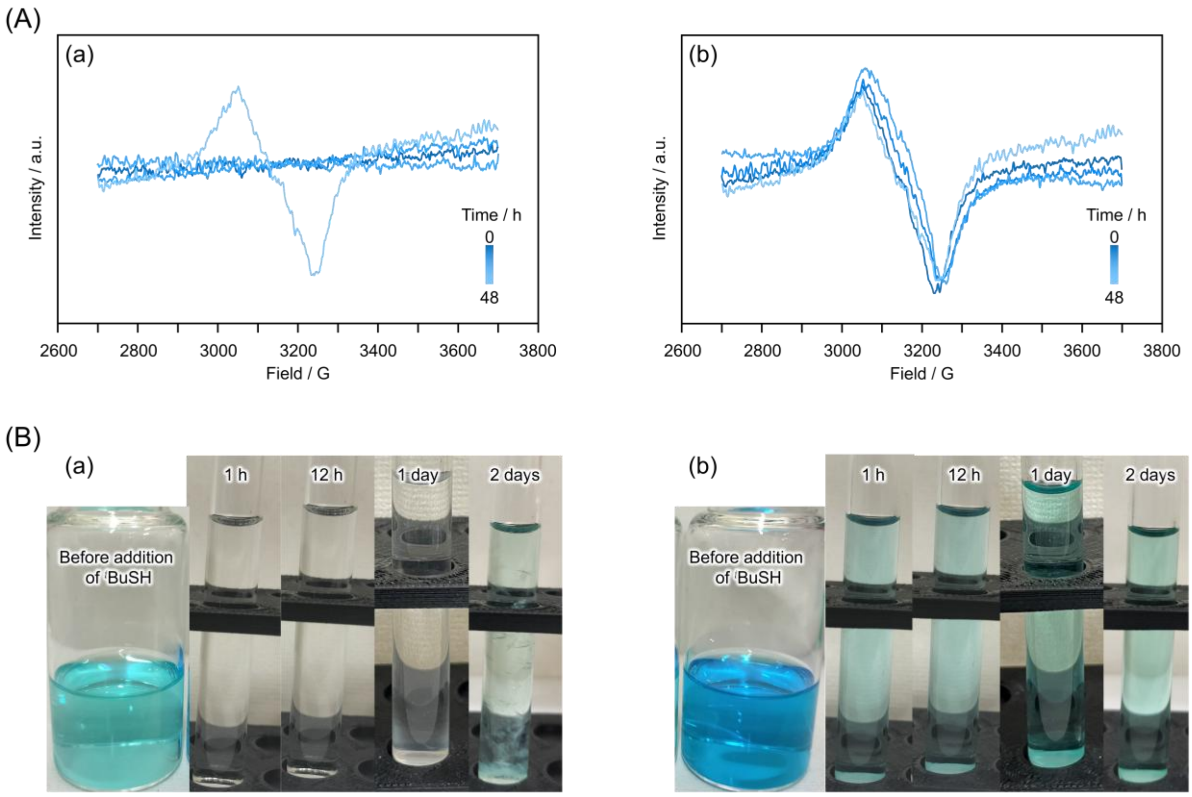

Figure S10. (A) ESR spectra and (B) photographs of reaction solution of (a) mixture of $Cu(NO_3)_2$ and ${}^tBuSH$, and (b) mixture of $Cu(NO_3)_2$, ${}^tBuSH$, and Ag(TFA) as a function of reaction time. These characteristic signals in ESR spectra originate from blue Cu^{2+} ($3d^94s^0$ electron configuration), while colorless Cu^+ ($3d^{10}4s^0$ electron configuration) shows no ESR signal. In (a), the color of the solution suddenly changed from blue to colorless after the addition of thiol, indicating that the reduction of Cu^{2+} (Cu^{2+} (blue) $\rightarrow$ Cu^+ (colorless)) occurred. 2 days later, the solution turned blue and the ESR signal appeared. This indicates that the oxidation of Cu^+ (Cu^+ (colorless) $\rightarrow$ Cu^{2+} (blue)) is slowly occurring. On the other hand, in (b), the solution remained blue and the Cu^{2+} derived signal was observed from the early stage of the reaction. This suggests that the oxidation of Cu^+ was accelerated by the addition of Ag^+.

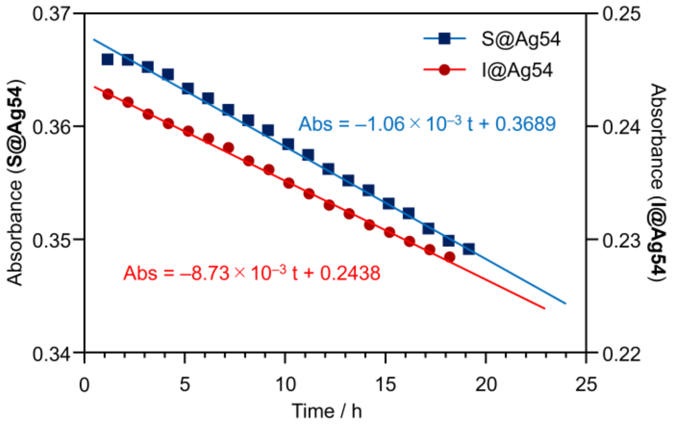

Figure S11. Time dependence of absorbance of **X@Ag54** in toluene (60 °C) at 370 nm.

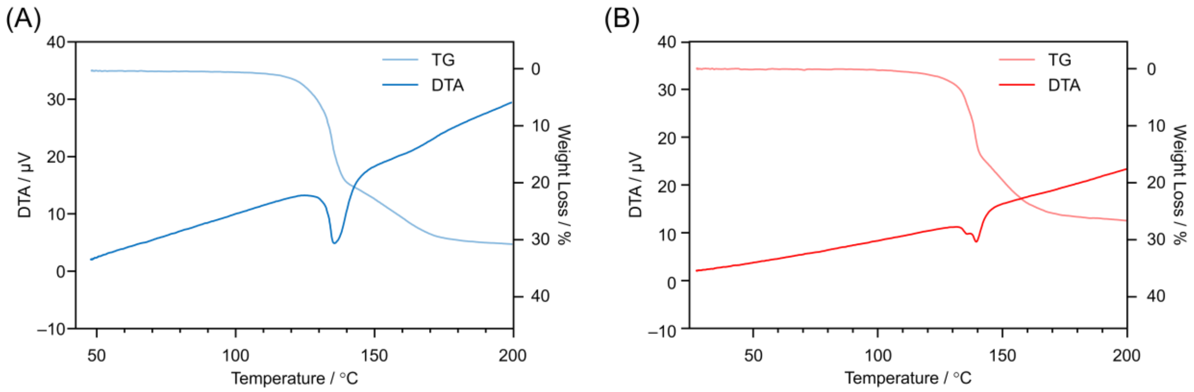

Figure S12. DTA curves of (A) **S@Ag54** and (B) **I@Ag54**. In these analyses, negative DTA peaks were observed at the temperatures corresponding to the dissociation of S–^{t}Bu and Ag–O_3S^tBu. This indicates that these dissociations are endothermic processes.

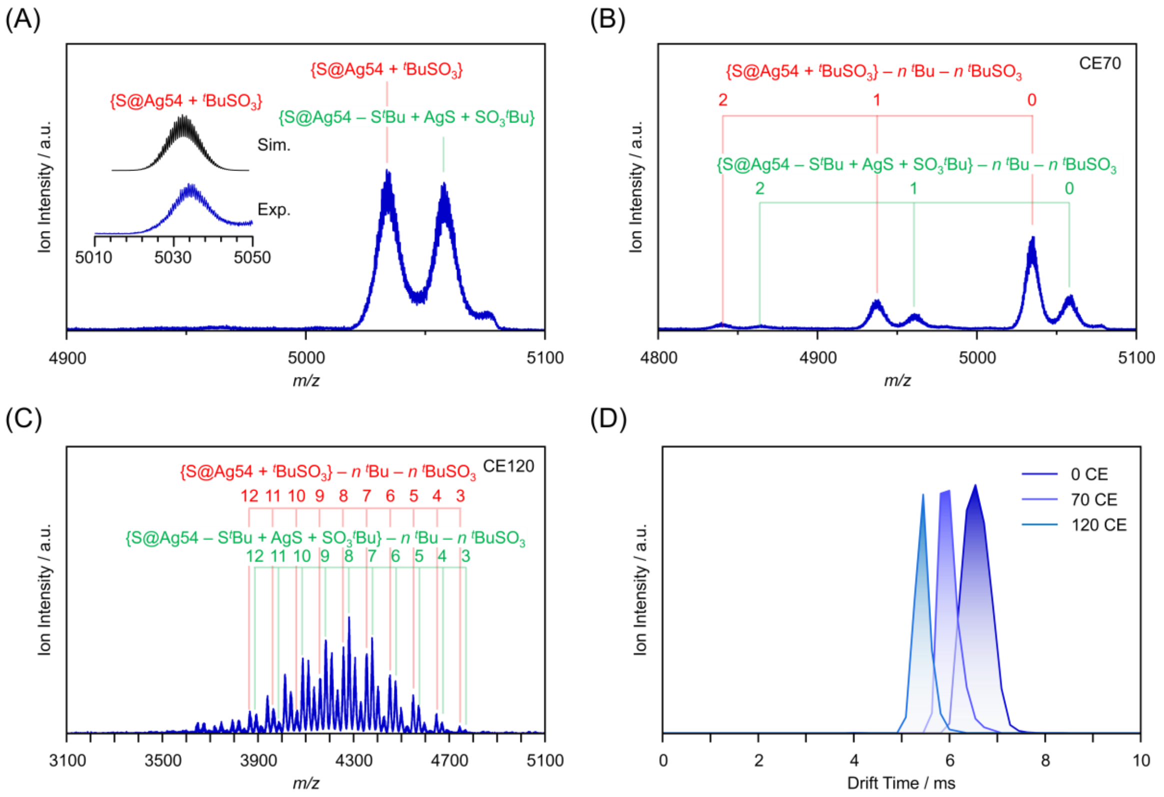

Figure S13. Positive-ion ESI-mass spectra of **S@Ag54** for CID. (A) Mass-selected molecular ion peak. (B) and (C) CID-MS spectra at collision energy (CE) of 70 and 120, respectively. In (A), since the *m*/*z*-difference between two main peaks were too close, both two-mother ions were analyzed by CID. (D) Ion-mobility spectra of **S@Ag54**. With the increase in CE, the ion mobility decreased. Based on a series of analyses (**Table S7**), the E_{50} values of **S@Ag54** and **I@Ag54** were evaluated as 74.2 CE and 78.9 CE, respectively, indicating that both **X@Ag54** possess similar stability against CID.

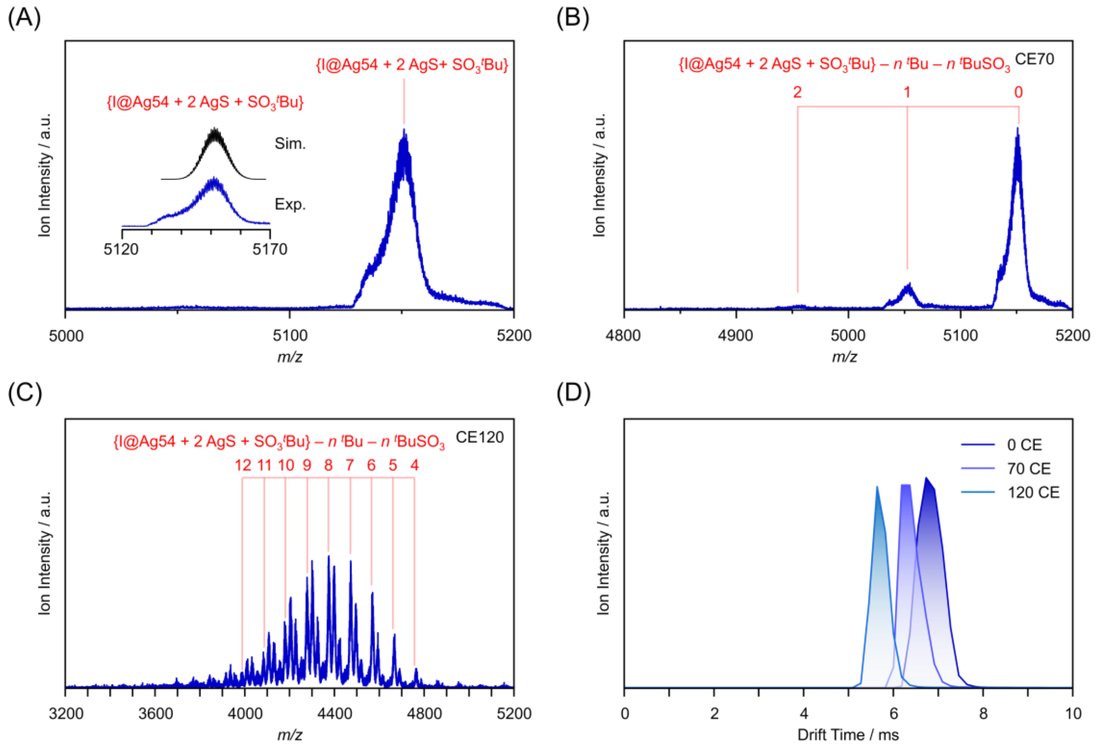

Figure S14. Positive-ion ESI-mass spectra of **I@Ag54** for CID. (A) Mass-selected molecular ion peak. (B), (C) CID-MS mass spectra at CE of 70 and 120, respectively. In (A), the isotope pattern was distorted because of mass-selection filter. (D) Ion-mobility spectra of **I@Ag54**. With the increase in CE, the ion mobility decreased. Based on a series of analyses (**Table S8**), the E_{50} values of **S@Ag54** and **I@Ag54** were evaluated as 74.2 CE and 78.9 CE, respectively, indicating that both **X@Ag54** possess similar stability against CID.

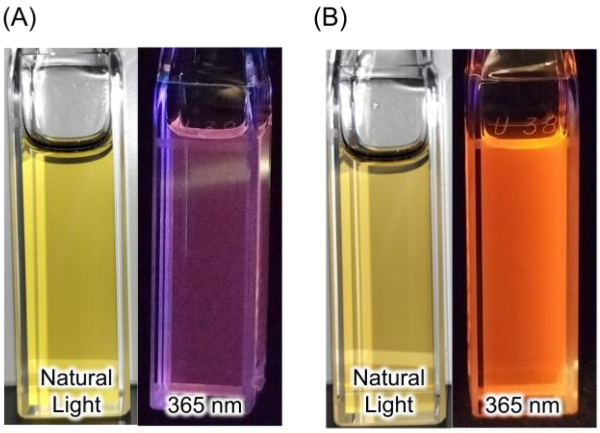

Figure S15. Photographs of solution of **X@Ag54** under natural light and 365 nm light. (A) **S@Ag54** and (B) **I@Ag54**. The emission spectra of **X@Ag54** at 365 nm excitation were also measured. Although the emission intensities were weaker than that otbained by 405 nm excitation, the emission spectrum showed the same feature as that otbained by 405 nm excitation.

S7. References

[1] A. Naumkin, A. Kraut-Vass, S. Gaarenstroom, C. Powell; **2023**; *NIST X-ray Photoelectron Spectroscopy Database*; 5.0; 10.18434/T4T88K.

[2] J. Roy, P. Chakraborty, G. Paramasivam, G. Natarajan, T. Pradeep, *Phys. Chem. Chem. Phys.* **2022**, *24*,2332.

[3] B. Valeur, M. Berberan-Santos, *Molecular Fluorescence: Principles and Applications*, 2nd ed., Wiley-VCH, Singapore, **2013**.

[4] K. Rurack, M. Spieles, *Anal. Chem.* **2011**, *83*,1232.

[5] M. J. Frisch, G. W. Trucks, H. B. Schlegel, G. E. Scuseria, M. A. Robb, J. R. Cheeseman, G. Scalmani, V. Barone, G. A. Petersson, H. Nakatsuji, X. Li, M. Caricato, A. V. Marenich, J. Bloino, B. G. Janesko, R. Gomperts, B. Mennucci, H. P. Hratchian, J. V. Ortiz, A. F. Izmaylov, J. L. Sonnenberg, Williams, F. Ding, F. Lipparini, F. Egidi, J. Goings, B. Peng, A. Petrone, T. Henderson, D. Ranasinghe, V. G. Zakrzewski, J. Gao, N. Rega, G. Zheng, W. Liang, M. Hada, M. Ehara, K. Toyota, R. Fukuda, J. Hasegawa, M. Ishida, T. Nakajima, Y. Honda, O. Kitao, H. Nakai, T. Vreven, K. Throssell, J. A. Montgomery Jr., J. E. Peralta, F. Ogliaro, M. J. Bearpark, J. J. Heyd, E. N. Brothers, K. N. Kudin, V. N. Staroverov, T. A. Keith, R. Kobayashi, J. Normand, K. Raghavachari, A. P. Rendell, J. C. Burant, S. S. Iyengar, J. Tomasi, M. Cossi, J. M. Millam, M. Klene, C. Adamo, R. Cammi, J. W. Ochterski, R. L. Martin, K. Morokuma, O. Farkas, J. B. Foresman, D. J. Fox, Gaussian 16 Rev. B.01, Program for the computational chemistry and electronic structure modeling, Gaussian, Inc., Wallingford (USA), **2016**

[6] P. J. Hay, W. R. Wadt, *J. Chem. Phys.* **1985**, *82*,299.

[7] M. M. Francl, W. J. Pietro, W. J. Hehre, J. S. Binkley, M. S. Gordon, D. J. DeFrees, J. A. Pople, *J. Chem. Phys.* **1982**, *77*,3654.

[8] N. J. Turro, V. Ramamurthy, J. C. Scaiano, *Modern Molecular Photochemistry of Organic Molecules*, Wiley-VCH, Singapore, **2011**.

[9] N. N. Greenwood, A. Earnshaw, *Chemistry of the Elements*, 2nd ed., Butterworth-Heinemann, Oxford, **1997**.

[10] a) M. Volmer, M. Stratmann, H. Viefhaus, *Surf. Interface Anal.* **1990**, *16*,278; b) L. J. Gerenser, K. E. Goppert-Berarducci, R. C. Baetzold, J. M. Pochan, *J. Chem. Phys.* **1991**, *95*,4641.
